# Proteome Profiling of Recombinant DNase Therapy in Reducing NETs and Aiding Recovery in COVID-19 Patients

**DOI:** 10.1016/j.mcpro.2021.100113

**Published:** 2021-06-15

**Authors:** Jane Fisher, Tirthankar Mohanty, Christofer A.Q. Karlsson, S. M. Hossein Khademi, Erik Malmström, Attila Frigyesi, Pontus Nordenfelt, Johan Malmstrom, Adam Linder

**Affiliations:** 1 Division of Infection Medicine, Department of Clinical Sciences Lund, Faculty of Medicine, Lund University, Lund, Sweden; 2 Division of Anaesthesia and Intensive Care, Department of Clinical Sciences Lund, Faculty of Medicine, Lund University, Lund, Sweden

**Keywords:** AF, Alexa Fluor, ARDS, acute respiratory distress syndrome, CF, cystic fibrosis, COT, conventional oxygen therapy, COVID-19, coronavirus disease 2019, DAPI, 4,6 diamidino-2-phenylindole, DIA-MS, data-independent acquisition MS, ECMO, extracorporeal membrane oxygen therapy, FDR, false discovery rate, FiO_2_, fraction of inspired oxygen, GGO, glass ground opacity, HFNO, high-flow nasal oxygenation, iRT, indexed retention time peptides, NE, neutrophil elastase, NET, neutrophil extracellular trap, PADI-4, peptidyl arginine deiminase IV, rhDNase, recombinant human DNase I, SARS-CoV2, severe acute respiratory syndrome coronavirus 2, SpO_2_, the oxygen saturation as measured by pulse oximetry

## Abstract

Severe coronavirus disease 2019 (COVID-19) can result in pneumonia and acute respiratory failure. Accumulation of mucus in the airways is a hallmark of the disease and can result in hypoxemia. Here, we show that quantitative proteome analysis of the sputum from severe patients with COVID-19 reveal high levels of neutrophil extracellular trap (NET) components, which was confirmed by microscopy. Extracellular DNA from excessive NET formation can increase sputum viscosity and lead to acute respiratory distress syndrome. Recombinant human DNase (Pulmozyme; Roche) has been shown to be beneficial in reducing sputum viscosity and improve lung function. We treated five patients pwith COVID-19 resenting acute symptoms with clinically approved aerosolized Pulmozyme. No adverse reactions to the drug were seen, and improved oxygen saturation and recovery in all severely ill patients with COVID-19 was observed after therapy. Immunofluorescence and proteome analysis of sputum and blood plasma samples after treatment revealed a marked reduction of NETs and a set of statistically significant proteome changes that indicate reduction of hemorrhage, plasma leakage and inflammation in the airways, and reduced systemic inflammatory state in the blood plasma of patients. Taken together, the results indicate that NETs contribute to acute respiratory failure in COVID-19 and that degrading NETs may reduce dependency on external high-flow oxygen therapy in patients. Targeting NETs using recombinant human DNase may have significant therapeutic implications in COVID-19 disease and warrants further studies.

## Highlights

• High levels of neutrophil extracellular traps (NETs) in the sputum of severe COVID-19 patients.
• Recombinant human DNase decreased NETs in sputum.
• Reduced NETs were associated with recovery and improved oxygenation.
• Mass spectrometry analyses of plasma and sputum indicate resolution of inflammation.

## Graphical abstract

**Figure:**
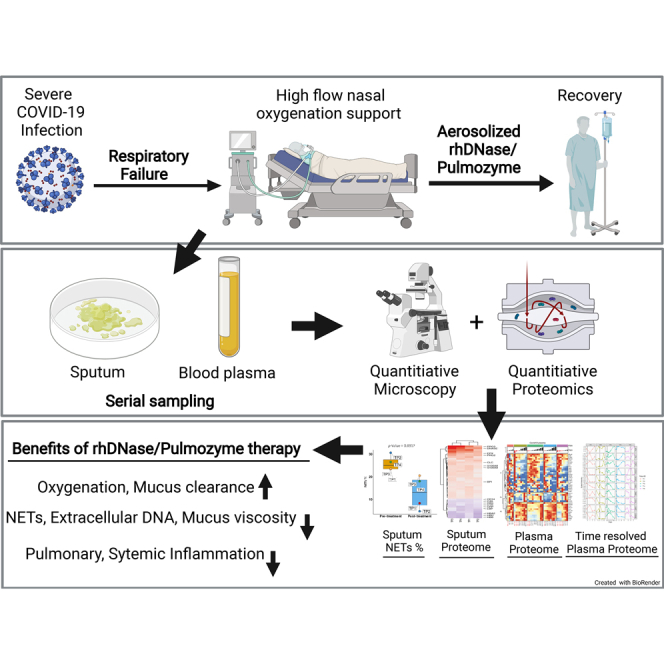

## In Brief

Neutrophils contribute to the extracellular DNA pool by forming neutrophil extracellular traps (NETs), which cause sputum thickening, pulmonary inflammation, and hindrance to gaseous exchange during infections. Here, we demonstrate the presence of NETs in sputum from severe COVID-19 patients using mass spectrometry and immunofluorescence analyses. Treatment with clinically approved recombinant human DNase reduced NETs and was associated with improved recovery and reduced inflammation. Targeting NETs using DNase may have significant therapeutic implications in COVID-19 disease and warrants further studies.

Coronavirus disease 2019 (COVID-19), the pandemic disease caused by the novel coronavirus severe acute respiratory syndrome coronavirus 2 (SARS-CoV2) (previously named 2019-nCoV, where “n” is for novel and “CoV” is for coronavirus), causes symptoms with severity ranging from a mild cold to severe pneumonia and acute respiratory distress syndrome (ARDS) that in some cases is fatal (1, 2, 3). The World Health Organization estimates that 15% of patients will have severe disease, 5% will have critical disease, and 3 to 4% will succumb to the disease (4). Patients with severe COVID-19 frequently develop ARDS and respiratory failure, characterized by hypoxemia, neutrophilia, pulmonary neutrophil infiltration, fibrin deposition, and buildup of thick mucus in the bronchi and bronchiectasis (5). Severely ill patients exhibit labored breathing and often require oxygen therapy through high-flow nasal oxygenation (HFNO), mechanical ventilation, or extracorporeal membrane oxygen therapy (ECMO) (6). However, these strategies have limitations because of harmful side effects (7) and an insufficient supply of ventilators (8).

ARDS in patients with COVID-19 is characterized by damaged alveoli, edema, hemorrhage, and intra-alveolar fibrin deposition (9). This causes a hazy appearance of blood vessels and airway structures when viewed using computerized tomography imaging, and this phenotype is termed glass ground opacity (GGO) (10). Hemorrhage, plasma leakage, and pulmonary neutrophil infiltration (3, 9) can cause the buildup of gelatinous and highly viscous sputum, which in turn produces the GGO phenotype in COVID-19 lungs (5). So far, molecular composition of sputum from patients with COVID-19 has remained uncharacterized. However, similar symptoms are seen in lungs of patients with ARDS and cystic fibrosis (CF), where neutrophil influx, acute phase plasma proteins, and inflammatory cytokines are present in sputum (11, 12, 13).

Sputum in ARDS and CF is highly complex and apart from proteins also contains abundant extracellular DNA, which causes mucus thickening. In fact, previous reports have shown that extracellular DNA increases mucus viscosity by 30% in CF (14), and inability to clear sputum from airways can lead to exacerbations and respiratory hypoxemia (15). During ARDS and CF, neutrophils can directly contribute to the extracellular DNA pool by forming neutrophil extracellular traps (NETs) (16, 17). NETs consist of extracellular DNA bound to neutrophil granule proteins and are released in response to bacteria (18) as well as some viruses (19). DNA decondensation preceding NET formation requires myeloperoxidase, neutrophil elastase (NE), and peptidyl arginine deiminase IV (PADI-4) activity (20). PADI-4 is known to catalyze arginine residues to citrulline in histones (21) and other granule proteins during NET formation (22, 23). NETs are hypothesized to aid the immune response by immobilizing and neutralizing virus particles (19, 20). Knockout of PADI-4 did not worsen experimental influenza (24), suggesting that NETs are not always an essential part of the immune response to viral infections. NETs can be cytotoxic to endothelial and lung epithelial cells (25) and can induce clot formation leading to vascular occlusion in the lungs (26), suggesting that a dysregulated NET response in the lungs can lead to significant damage. It has been hypothesized that NETs may play a role in COVID-19 (27, 28), and markers of NETs have been detected in the plasma of patients with COVID-19 (29).

In CF, NETs can be degraded using DNase I. Preclinical studies have suggested that removal of NETs using DNase is beneficial in both bacterial (30) and viral (31, 32) diseases. Production of highly viscous sputum may cause ARDS in COVID-19. Accumulation of thick sputum in the airways can interfere with the gaseous exchange, which in turn leads to hypoxemia (33), increased use of mechanical ventilation, and an increased risk of mortality. Therefore, improving mucus clearance from airways by altering sputum viscosity may improve pulmonary oxygenation and prevent development of ARDS. This strategy may also reduce dependency on mechanical ventilation and reduce the risk of mortality during COVID-19. Recombinant human DNase I (rhDNase) could potentially be used to target dysregulated NET formation in severe COVID-19. rhDNase (Pulmozyme; Roche) is currently used safely in humans to reduce mucus thickness in CF (34). However, the current understanding of alterations in the composition of sputum and blood plasma proteome during SARS-CoV2 pathogenesis remains limited.

In this study, we applied SWATH-like data-independent acquisition MS (DIA-MS) (35) to examine the sputum and blood plasma proteome from patients with COVID-19. We found neutrophil/NET-derived proteins, including neutrophil granule proteins and citrullinated proteins, and acute phase proteins associated with exaggerated inflammation in sputum. Immunofluorescence analysis of sputum from COVID-19 revealed the presence of NETs in the sputum that could be degraded using DNase I *ex vivo*. Furthermore, to gain preliminary insights into the action of rhDNase in improving respiratory function, a small cohort of severely ill patients with COVID-19 were treated with rhDNase followed by molecular characterization of blood plasma and sputum using immunofluorescence and proteomics analysis.

## Experimental Procedures

### Patient Enrolment and Sample Collection

The sample collection was approved by the Lund University Local Ethics Committee (application number: 2016/39) and was in accordance with the ethical principles in the Helsinki declaration. Informed consent was collected from all participants or next of kin. We enrolled ten patients in the study from March 17 to April 12, 2020. The included patients were admitted to the Clinic for Infectious Diseases in Lund with confirmed COVID-19 by positive SARS-CoV2 revere transcription-polymerase chain reaction assay and a need for respiratory support to maintain an oxygen saturation >93%. Patients who were treated with rhDNase were followed with serial sampling until hospital discharge. Demographic and clinical data were collected retrospectively from the patients' charts. Venous EDTA-blood (K2EDTA; BD Vacutainer;10 ml, BD Biosciences) was collected once or twice prior to rhDNase treatment and once daily following rhDNase treatment. Sputum was collected whenever it was possible by spontaneous production (coughing). Not all patients were able to expectorate sputum, and therefore they were excluded from sputum NET analyses where relevant. Platelet poor plasma was collected by centrifuging EDTA blood at 1800*g* for 10 min at room temperature. Sputum samples were collected in 70 ml multipurpose polypropylene sterile containers without any additives. All samples were processed within 4 h after collection from patients. The interval between processing times and freezing of samples was limited to a maximum of 15 min for plasma and 30 min for sputum to minimize variability.

### Healthy Controls

Blood and sputum were collected from four donors who were not exhibiting any respiratory symptoms and therefore were assumed to be SARS-CoV2 negative. We cannot rule out asymptomatic infections in these donors. Collection of blood from healthy donors was approved by the Lund University Local Ethics Committee (application number: 2013/728).

### Treatment

All patients were given standard clinical care for their condition. Three SARS-CoV2–positive patients were analyzed for sputum NETs but were not treated with rhDNase. Five SARS-CoV2–positive patients (referred to as patients TP 1–5) treated with rhDNase (Pulmozyme), administered by the decision of the treating physician as “off-label” use. Four of these patients (TP 1–4) were able to expectorate sputum before and after treatment and were analyzed for NETs. Treatment with rhDNase was given *via* nebulizer at a dose of 2.5 mg twice daily until the treating physician's decision to stop treatment. All patients were treated with oxygen therapy either by conventional oxygen therapy (COT) or HFNO therapy at time of treatment start. The intervention was not randomized, and patients and clinicians were not blinded.

### *Ex vivo* DNase Treatment of Sputum Sample

Sputum sample from a patient with COVID was treated with 10 units of rhDNase (Abcam) for 10' at 37 °C. An aliquot of the same sample was treated the same way but without addition of DNase I. Samples were cytocentrifuged and then prepared for immunostaining as described later.

### Calculated Variables

Estimated mean arterial pressure was calculated by doubling the diastolic pressure and adding the systolic pressure and dividing this sum by three. The fraction of inspired oxygen (FiO_2_) when patients were receiving COT *via* nasal cannula or face mask was estimated by multiplying the oxygen flow rate by 0.04 and adding this number to 0.2 (36). When patients were receiving HFNO therapy, the FiO_2_ was estimated by the oxygen percentage set on the blender. Because arterial oxygen partial pressure/FiO_2_ was not measured in these patients, the SpO_2_ (the saturation of oxygen as measured by pulse oximetry)/FiO_2_ ratio was calculated as a surrogate (37).

### Sputum NET Analysis

Sputum was immediately fixed with 4% paraformaldehyde (Sigma–Aldrich) in PBS (Sigma–Aldrich) at 4 °C for 1 h. About 10 μl of the fixed sputum mixture was diluted with 500 μl of PBS and cytocentrifuged for 10 min at 2000 rpm onto glass slides. Samples were permeabilized with 0.5% Triton X-100 (Sigma–Aldrich) for 20 s and then blocked with blocking buffer (5% goat serum [BioWest] with 0.05% Tween-20 [MP Biologicals] in PBS) at 37 °C for 30 min. Samples were stained with rabbit-antihuman NE antiserum (Dako; 1:500 dilution) in blocking buffer at 4 °C overnight, then stained with secondary Alexa Fluor (AF)-647–conjugated goat-anti-rabbit Fab2’ antibody fragment (1:1000 dilution; Life Technologies) in blocking buffer at 37 °C for 1 h. Samples were washed three times with PBS and coverslips (Menzel-Glaser; #1.5 thickness) were mounted on the samples using mounting media with ProLong Gold antifade reagent with 4,6 diamidino-2-phenylindole (DAPI; Life Technologies) and cured overnight.

For 3D super-resolution imaging, samples were stained with rabbit-antihuman NE antiserum and AF-568–conjugated goat-anti-rabbit secondary antibody. DNA was stained with 5 μM DRAQ5 for 30 min at room temperature, and coverslips were mounted with ProLong Gold antifade reagent (Life Technologies).

### Fast Staining Protocol

Some samples were prepared for same-day analysis. The protocol for NET analysis was followed as aforementioned with some changes. Fixation was done for a minimum of 30 min at 4 °C. Blocking was done for a minimum of 15 min at 37 °C. Primary and secondary antibody incubation was done for a minimum of 15 min at room temperature. Samples were washed as normal, and coverslips were mounted with a drop of mounting media with DAPI. Clear nail polish was applied to the edges of the coverslip and was then allowed to dry. Samples were imaged directly.

### Widefield Image Acquisition

All widefield images were collected with a Nikon Ti-2 inverted microscope equipped with a 20×/0.75 or a 40×/0.95 (magnification/numerical aperture) objective and the Perfect Focus System for maintenance of focus over time. Fluorophores were excited with a Lumencor SpectraX light engine. AF-594 was excited with the 57- nm line from a 330 mW light-emitting diode source and collected with a DM593 dichroic mirror and a 624/40 nm emission filter. DAPI was excited with the 395-nm line from a 295 mW light-emitting diode source and collected with a DM409 dichroic mirror and a 447/60 nm emission filter. Images were acquired with a Nikon DS-Qi2 sCMOS camera controlled with NIS Elements AR software. Multiple stage positions were collected using a motorized piezo stage. Whole slide scanning was performed using NIS Elements JOBS to acquire 6 × 6 20× images to cover the whole sample circle. Frames were stitched using 5% overlap at the edges and automatic shading correction.

### Widefield Image Analysis and Quantification

All unstitched frames from each sample were quantified using the NETQUANT app (version 1.3) in MATLAB (version 2019b) (38). The software uses thresholds for NET criteria that are set by the user, and for the analysis applied here, the following are the thresholds: cell area fold increase 3.50; nuclei deformation 0.30; and DNA/NET area 0.8 or 2.0. Elastase staining in the samples was heterogeneous, likely because of varying amounts of neutrophil activation between patients, making it necessary to apply two different segmentation settings for the elastase channel. The “Global” option applies Otsu's method (39), where a segmentation threshold is selected that minimizes the intraclass variance of black and white pixels. The “Adaptive” option uses Bradley's method to calculate a locally adaptive threshold using local first-order statistics around each pixel (Bradley and Roth, 2007). For the DNA channel, adaptive segmentation was used with a sensitivity of 0.2. Representative images were processed in Fiji (40).

### Super-Resolution 3D Structured Illumination Microscopy

All super-resolution images were collected with an N-SIM E system on Nikon Ti-E inverted microscope equipped with a Plan Apochromat Lambda 100×/1.49 (magnification/numerical aperture). AF-594 was excited with the 561-nm line from a laser source and collected with an N-SIM561 filter. DRAQ5 was excited with the 640-nm line from a laser source and collected with an N-SIM640 filter. Z-series optical sections were collected with a step size of 0.3 microns. Images were acquired with a Hamamatsu Orca Flash 4.0 sCMOS camera controlled with NIS Elements AR software. The SIM images were reconstructed with the NIS-elements AR algorithm for reconstruction.

### MS Sample Preparation

To liquefy the sputum, all sputa were treated with 15 mM Tris(2-carboxyethyl)phosphine hydrochloride (Sigma) for 10 min at room temperature. These were then centrifuged at 500*g* for 10 min at room temperature to obtain a supernatant and pellet. The pellet was resuspended in 100 μl 0.2% RapiGest SF surfactant (Waters) and boiled for 10 min and cooled on ice for 10 min. An equal volume of 8 M urea in 0.1 M ammonium bicarbonate (Sigma) solution was added to the samples. BCA (Pierce) was then performed on the samples to estimate protein concentration, and 50 μg of protein was taken for digestion.

Blood from EDTA tubes was processed by centrifugation for 10 min at 500*g* at room temperature to obtain the buffy coat. The supernatant was taken into fresh tubes and spun for 10 min at 2000*g* to remove platelets. 100 μl of plasma was diluted 1:10 by adding 900 μl of 8 M urea and 0.1 M ammonium bicarbonate solution and stored at −20 °C. 10 μl of the diluted plasma was digested.

Proteins were reduced with 5 mM Tris(2-carboxyethyl)phosphine hydrochloride, pH 7.0 for 45 min at 37 °C, and alkylated with 25 mM iodoacetamide (Sigma) for 30 min followed by dilution with 100 mM ammonium bicarbonate to a final urea concentration below 1.5 M. Proteins were digested by incubation with trypsin (1/100, w/w, Sequencing Grade Modified Trypsin, Porcine; Promega) for at least 9 h at 37 °C. Digestion was stopped using 5% trifluoracetic acid (Sigma) to pH 2 to 3. The peptides were cleaned up by C18 reversed-phase spin columns as per the manufacturer's instructions (Silica C18 300 Å Columns; Harvard Apparatus). Solvents were removed using a vacuum concentrator (Genevac, miVac) and were resuspended in 50 μl HPLC-water (Fisher Chemical) with 2% acetonitrile and 0.2% formic acid (Sigma). Samples were spiked with indexed retention time peptides (iRT) peptides prior to MS analysis.

### LC–MS/MS Analysis

All peptide analyses were performed on a Q Exactive HF-X mass spectrometer (Thermo Fisher Scientific) connected to an EASY-nLC 1200 ultra-HPLC system (Thermo Fisher Scientific). Peptides were trapped on precolumn (PepMap100 C18 3 μm; 75 μm × 2 cm; Thermo Fisher Scientific) and separated on an EASY-Spray column (ES803, column temperature 45 °C; Thermo Fisher Scientific). Equilibrations of columns and sample loading were performed per manufacturer's guidelines. Solvent A was used as stationary phase (0.1% formic acid), and solvent B (mobile phase; 0.1% formic acid, 80% acetonitrile) was used to run a linear gradient from 5% to 38% over 90 or 120 min at a flow rate of 350 nl/min. For data-dependent acquisition, one full MS scan (resolution 60,000 at 200 *m*/*z*; mass range 350–1650 *m*/*z*) was followed by MS/MS scans (resolution 15,000 at 200 *m*/*z*) of the 20 most abundant ion signals (TOP20). The precursor ions were isolated with 1.6 *m*/*z* isolation width and fragmented using higher-energy collisional-induced dissociation at a normalized collision energy of 27. Charge state screening was enabled, and unassigned or singly charged ions were rejected. The dynamic exclusion window was set to 10 s. Only MS precursors that exceeded a threshold of 8e3 were allowed to trigger MS/MS scans. The ion accumulation time was set to 100 ms (MS) and 30 ms (MS/MS) using an automatic gain control target setting of 2e5 (MS and MS/MS). *m*/*z* extraction window was set at 20 ppm. The 44 variable windows DIA acquisition method is described by Bruderer *et al*. (41). For DIA analysis, the gradient length was set to 120 min for sputum and 90 min for plasma respectfully. The MS data files were analyzed with FragPipe (42) and OpenSWATH (43) in the context of the human reference proteome (EMBL-EBI RELEASE 2019_04).

### Spectral Library Generation

Peptides from sputum or plasma pre-rhDNase treatment were pooled and fractionated (n = 8) with off-line high-pH reversed-phase chromatography (Thermo Fisher Scientific) respectfully. The fractions together with unfractioned samples were analyzed with DDA (totally 57 injections), and the resulting data searched against the human reference proteome acquired from EMBL-EBI (release 201904) merged with SARS-CoV-2 proteome (downloaded from Uniprot.org; March 28, 2020) consisting of totally 20,888 target entries using FragPipe (version 13.2) and MSFragger (version 2.4) search engine with FragPipe default settings for modifications and “closed search” (1% protein false discovery rate [FDR]). SpectraST (version 5.0) was used for consensus spectra building and msproteomictools (version 0.11) for iRT alignment (high pH fractions were spiked with PROCAL peptides (44) and other samples with iRT peptides (45)). Assays were selected by restricting the transitions by DIA acquisition windows (exclusion of fragment ions falling into the precursor isolation window); precursor and product mz threshold of 0.025 Th; and maximal number of transitions set to eight by OpenSWATH (docker hub openswath/openswath:0.2.1). Decoy peptides and assays were generated with OpenSwath; counts of “target:decoy” features are n protein groups = “4592:4455,” n precursors = “37,247:34,807,”and n stripped peptide sequences = “27,142:28,435.”

For querying citrulline residues with DIA, an additional variable modification (+0.984016 Da) on arginine residues was used in a separate FragPipe search. The raw files and parameters for library generation post the search was identical to the aforementioned workflow. The citrulline library was used for generating supplemental Figure S1 only.

### DIA Data Analysis

About 42 plasma and 25 sputum DIA data files were analyzed separately with OpenSWATH (docker hub openswath/openswath:0.2.1) with the following options: —readOptions workingInMemory; —sort_swath_maps; —rt_extraction_window 600; —mz_extraction_window 20 ppm; —use_ms1_traces; —mz_correction_function quadratic_regression_delta_ppm; —Scoring:TransitionGroupPicker:background_subtraction exact; —batchSize 2000. The extracted OpenSWATH data were statistically validated with Pyprophet (docker hub openswath/openswath:0.2.1) with score options on both ms1 and ms2; peptide and protein inferment with error-rate estimation on contexts run-specific, experiment-wide and global; data were exported with the option max_global_protein_qvalue set to *q* value of 0.01. The pyprophet output data table was feature aligned with the TRIC algorithm (PyPI package msproteomicstools 0.11.0) with the following parameters: “-realign_method lowess_cython, -max_rt_diff 60, -method LocalMST, -mst:useRTCorrection True, -mst:Stdev_multiplier 3.0, -target_fdr 0.01, -max_fdr_quality 0.05, and -alignment_score 0.001.”

Protein quantities were summed up by file and protein and then normalized to the total protein intensity per file. Protein groups were removed, and proteins were filtered for data completeness requiring quantification in at least ten files per sample. Regarding quantification of SARS-CoV-2 proteins, the library included four viral peptides, of which two were quantified in two different sputum samples and hence did not pass the data completeness filter.

## Results

### Characterization of Sputum Proteome in COVID-19 Disease

**Fig. 1:**
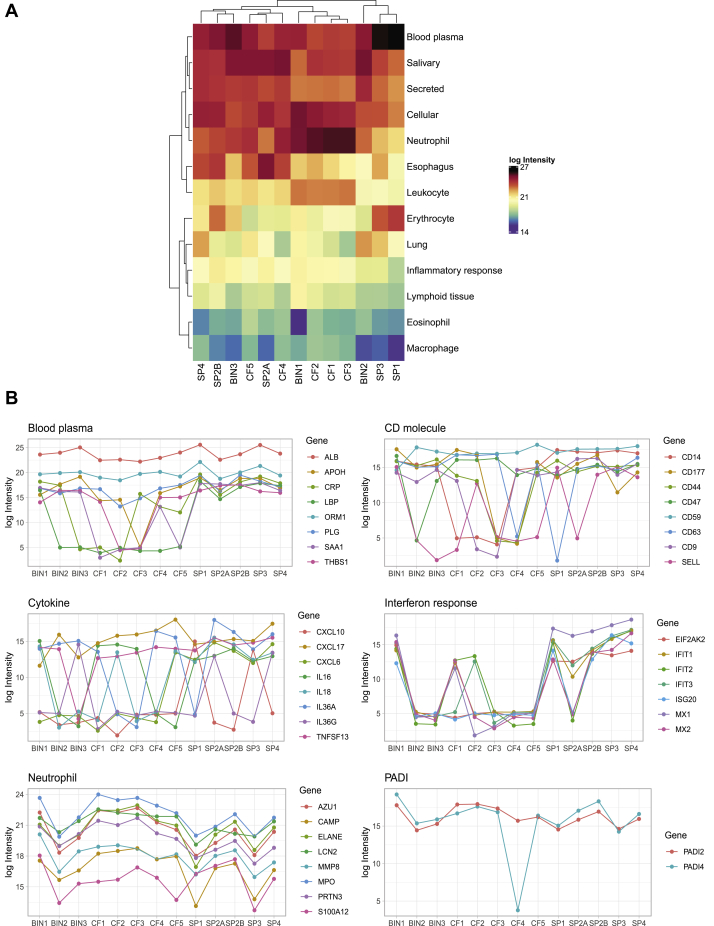
**Characterization of lung sputum from COVID-19 patients using MS-based proteomics.** Sputum from severely ill COVID-19 patients (SP1–4, n = 4), cystic fibrosis (CF1–5, n = 5), and bacterial infections (BIN1–3, n = 3) was collected and analyzed using MS. Two samples from a single patient (SP2A and SP2B), collected 2 days apart, were included in the analysis. About 2037 proteins were discovered in sputum with 1% FDR (supplemental Table S1). Proteins were then assigned manually to 13 protein classes using protein functions in Swiss-Uniprot and human protein atlas. *A*, heat map showing the log10 intensities of the assigned protein classes in lung sputum (n = 5) from four COVID-19 patients, CF (n = 5), and bacterial infections (n = 3). *B*, plots of individual protein intensities grouped by class in the different sputums. The mapped gene names of the proteins are indicated. FDR, false discovery rate.

Similar to CF and pulmonary bacterial infections, severe COVID-19 disease affects lungs and results in accumulation of gelatinous mucus in the airways (5). Expectoration of sputum allows the collection of accumulated material in the airways that can provide insights on the composition and inflammatory status of the mucus (46, 47). Here, we collected sputum from four severely ill patients with COVID-19 admitted in the clinic for infectious diseases at Skåne University hospital in Lund, Sweden, between March 17 and April 12, 2020 and compared them with sputa from CF (n = 5) and bacterial infections (n = 3). Quantitative DIA-MS analysis reveals that the sputum proteome is highly complex, variable, and comprises of more than 2000 unique proteins (Fig. 1*A*). In COVID-19, the most abundant proteins were both subtypes of immunoglobulin A and mucins, followed by blood plasma proteins, such as albumin, leukocyte proteins, and inflammatory/antiviral response proteins, such as interferon-induced proteins (supplemental Table 1). Some of the abundant and relevant protein groups include blood plasma proteins, cell surface markers, inflammatory cytokines, and interferon-induced proteins and are presented in Figure 1*B*. Upon comparison to sputum from other inflammatory conditions like bacterial infections and CF, a common set of proteins that include acute phase blood plasma proteins like albumin, alpha-1-acid glycoprotein 1, neutrophil proteins, and PADI enzymes were found to be elevated. These protein groups are associated with plasma leakage, inflammation, antiviral responses, and leukocyte influx and reflect the inflammatory state of severe COVID-19 infected airways. The high levels of several known neutrophil granule and cytosol proteins are likely because of pulmonary neutrophil influx (Fig. 1*B*). PADI-4 or protein-arginine deiminase type-4 is a neutrophil-enriched nucleus enzyme that catalyzes citrullination of histones and neutrophil proteins prior to NET release (22, 23). Interestingly, several neutrophil proteins and histones were citrullinated in COVID-19 (supplemental Fig. S1), possibly as a result of the detectable levels of PADI-4 or protein-arginine deiminase type-4 (48) (Fig. 1*B*). Taken together, these results indicate that the sputum is inflammatory active and that there are high levels of neutrophil and a substantial degree of NET-related proteins in COVID-19 sputum.

### NETs Are Present in the Sputum of Severely Ill Patients with COVID-19

**Fig. 2:**
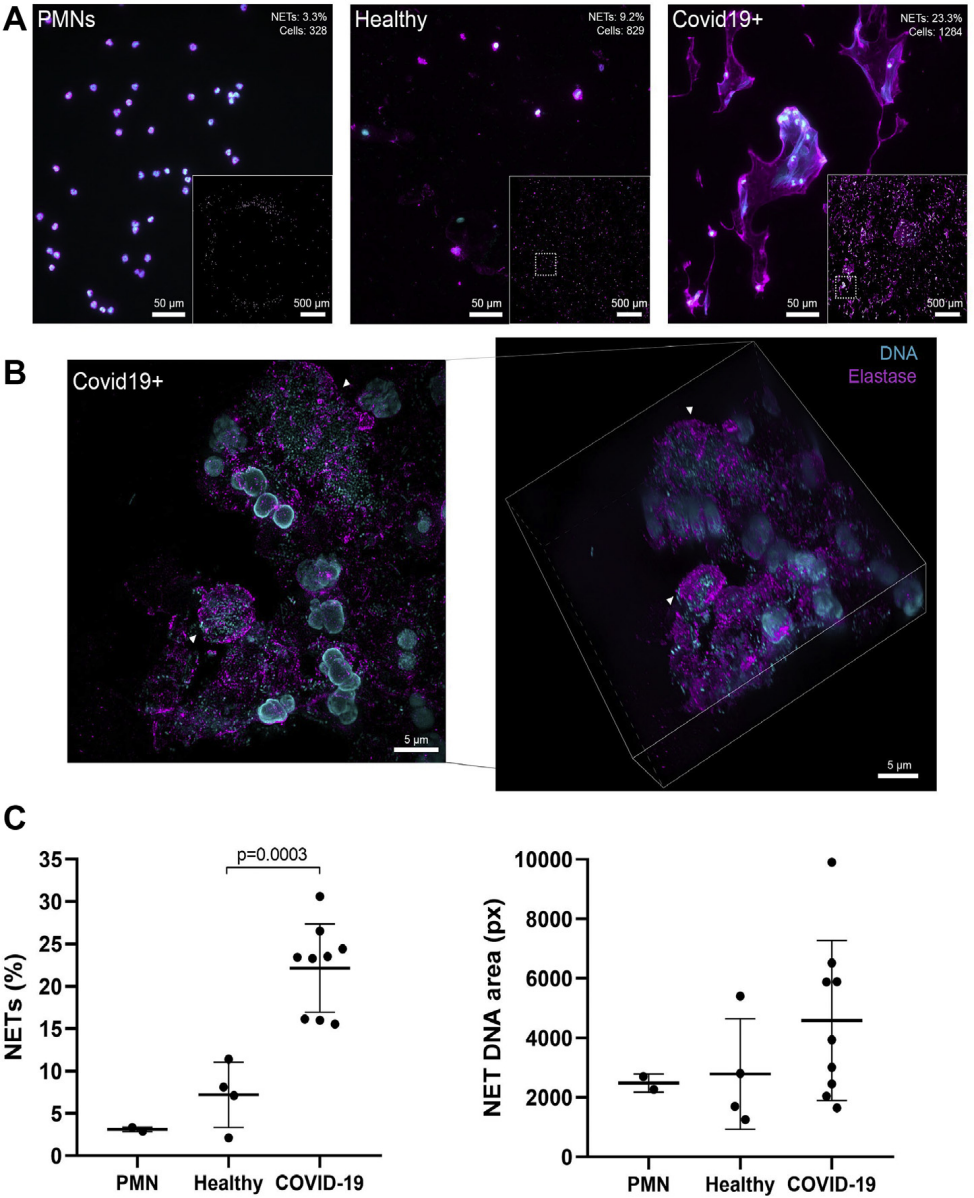
**NETs in the sputum of SARS-CoV2 positive who were not treated with rhDNase and healthy controls.***A*, widefield microscopy of neutrophil elastase (*magenta*, antielastase antibody) and DNA (*cyan*, DAPI) in sputum samples or purified neutrophils. Insets show stitched overview image from 6 × 6 images of the samples. Quantification of single cell–level NETs (percent of cells and number of cells) of each overview is shown in the top corner of representative images. *B*, super-resolution microscopy of neutrophil elastase (*magenta*, antielastase antibody) and DNA (*cyan*, DRAQ5) in a nontreated patient sample. *Left*, maximum intensity projection of structured illumination microscopy Z-stack. *Right*, isometric view of Z-stack. *Arrowheads* show either a fully formed NET (*top*) or a neutrophil with decondensed DNA undergoing NETosis (*bottom*). *C*, quantitative data of *A* are shown as the percentage of NET-positive cells and the corresponding average area that the DNA signals covers in each cell. Each point indicates quantification of 36 images from independent samples. *Lines* indicate the mean for each category. DAPI, 4,6 diamidino-2-phenylindole; NETs, neutrophil extracellular traps; rhDNase, recombinant human DNase I; SARS-CoV2, severe acute respiratory syndrome coronavirus 2.

Discovery of neutrophil proteins, PADI enzymes, citrullinated neutrophil proteins, and histones, all of which are associated with NET formation, prompted us to investigate whether NETs are present within the respiratory system of patients with COVID-19. We collected sputum from SARS-CoV2—positive patients with different disease severities admitted to the clinic for infectious diseases at Skåne University hospital in Lund, Sweden. Only nine patients were able to expectorate sputum. Using immunofluorescence microscopy against NE and DNA as described previously (30), and in agreement with the MS data, we readily found neutrophil infiltration and NETs in the sputum of patients with COVID-19 (Fig. 2*A*). Cells were found in various stages of NETosis (Fig. 2*B* and supplemental Movie S1), ranging from early-phase NETs (Fig. 2*B*, *bottom arrow*) to large, diffuse, and completely extracellular NET structures (Fig. 2*B*, *top arrow*). Image quantification revealed that on average 22% of cells formed NETs in COVID-19 sputum, whereas only 7% cells underwent NETosis in healthy sputum (*p* = 0.0003) (Fig. 2*C*). In contrast, the mean DNA size of NET-forming cells was not significantly larger in COVID-19 sputum compared with healthy sputum (average 4587 *versus* 2788 pixels/px; *p* = 0.230) (Fig. 2*C*). To determine the susceptibility of NETs in COVID-19 to DNase I, sputum from a patient with COVID-19 was treated with commercially available DNase I or left untreated *ex vivo*. DNase treatment resulted in the rapid degradation of NETs in the sample within 10 min in comparison to untreated sputum (supplemental Fig. S2). The DNase-treated sample also showed a marked reduction in turbidity and viscosity upon visual inspection.

### Treatment of Severely Ill COVID-19 Patients with rhDNase Was Associated with Improved Clinical Outcomes

**Fig. 3:**
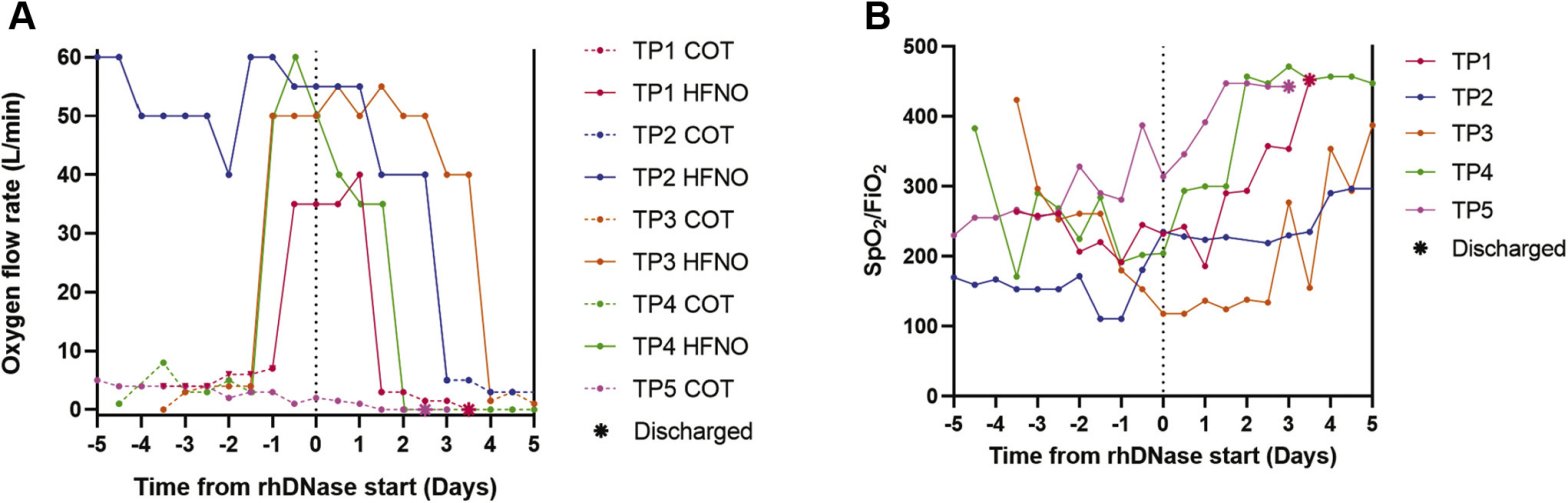
**Oxygen requirements and SpO**_**2**_**/FiO**_**2**_**ratios of patients receiving rhDNase.** Severely ill patients with COVID-19 that required high-flow nasal oxygen (HFNO) or conventional oxygen therapy (COT) (n = 5) were treated with aerosolized rhDNase (TP1–5). *Dashed vertical line* at the center of the graph denotes start of rhDNase treatment. Oxygen flow rate (*A*) and estimated SpO_2_/FiO_2_ ratio (*B*) of patients receiving HFNO therapy at the time of rhDNase treatment start. *Solid lines* are flow rate during HFNO, whereas *dashed lines* are flow rate during COT. *Asterisks* indicate hospital discharge. COVID-19, coronavirus disease 2019; FiO_2_, fraction of inspired oxygen; rhDNase, recombinant human DNase I; SpO_2_, the oxygen saturation as measured by pulse oximetry.

The marked reduction in turbidity and viscosity after DNase treatment indicates that aerosolized DNase could be used to degrade extracellular DNA, reduce sputum viscosity, and improve respiratory function. To test this notion, we treated five severely ill patients with COVID-19 admitted to the clinic for infectious diseases at Skåne University hospital in Lund, Sweden, with rhDNase. All patients at the time of treatment required external oxygen therapy (baseline characteristics of the patients receiving rhDNase therapy are presented in Table 1 and Table S1). COT or HFNO was administered to the patients prior to rhDNase therapy. Remarkably, within 4 to 15 days of rhDNase treatment, none of the patients went on to require intensive care unit admission or mechanical ventilation, and all were weaned off oxygen therapy. Oxygen demand for the four HFNO patients (patients 1–4; Fig. 3*A*) declined within 1 to 3 days after start of rhDNase administration, and SpO_2_/FiO_2_ ratio began to climb concurrently (Fig. 3*B*). All patients receiving rhDNase treatment recovered.

**Table 1:** Characteristics of treated patients

Characteristics	Values
Age; mean (SD)	66.4 ± 5.72
Males; n (%)	4 (80)
Illness before admission (days); mean (SD)	6 ± 4.5
Hospital stay (days); mean (SD)	26.8 ± 17.9
Comorbidities and previous medications, n (%)	
Hypertension	2 (40%)
Other cardiovascular	2 (40%)
ACE2 inhibitors	2 (40%)
Vital signs on admission; mean (SD)	
Pulse (beats/min)	96.6 ± 9.31
Respiratory rate (breaths/min)	21.2 ± 3.03
Temperature (°C)	37.32 ± 0.89
Mean arterial pressure (mm Hg)	75.2 ± 5.16
O_2_ saturation (%)	89.2 ± 5.35
Organ failures; n (%)	
Respiratory	5 (100%)
Kidney	4 (80)
Circulatory	2 (40)
Oxygen requirements	
Any external oxygen	
Number; n (%)	5 (100)
Days; mean (SD)	12 ± 7.87
HFNO	
Number; n (%)	4 (80)
Days; mean (SD)	6.25 ± 4.47

### Degradation of NETs and Sputum Proteome Reorganization after rhDNase Treatment

**Fig. 4:**
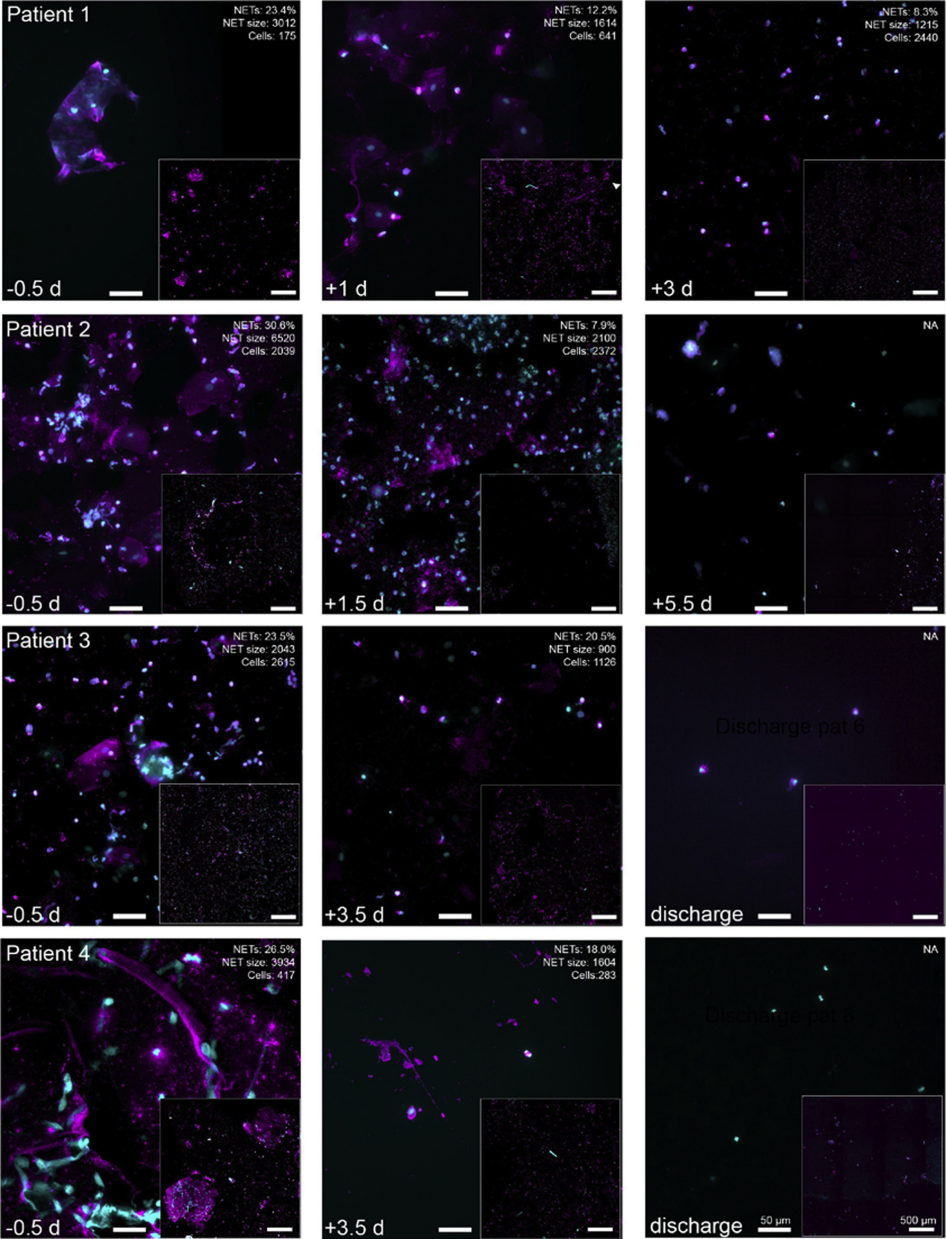
**NETs and neutrophil proteins in the sputum of patients receiving rhDNase.** Sputum could be collected from four of five patients who received rhDNase treatment. Widefield microscopy of neutrophil elastase (*magenta*, antielastase ab) and DNA (*cyan*, DAPI) in sputum samples from treated patients (TP 1–4). Insets show stitched overview image from 6 × 6 images of the samples. Quantification of single cell–level NETs (percent of cells, average NET size, and number of cells) of each overview is shown in the top corner of representative images. The scale bars are the same for each image and corresponds to 50 μm (high magnification) or 500 μm (overview inset), respectively. Day of. sputum sampling is indicated in the *bottom left corner*. For some images, there were too few cells (<100) identified for reliable quantification, and thus they were excluded from the analysis. The images shown in this figure are part of the NETs quantification in Figure 5, *A* and *B*. DAPI, 4,6 diamidino-2-phenylindole; NETs, neutrophil extracellular traps; rhDNase, recombinant human DNase I.

**Fig. 5:**
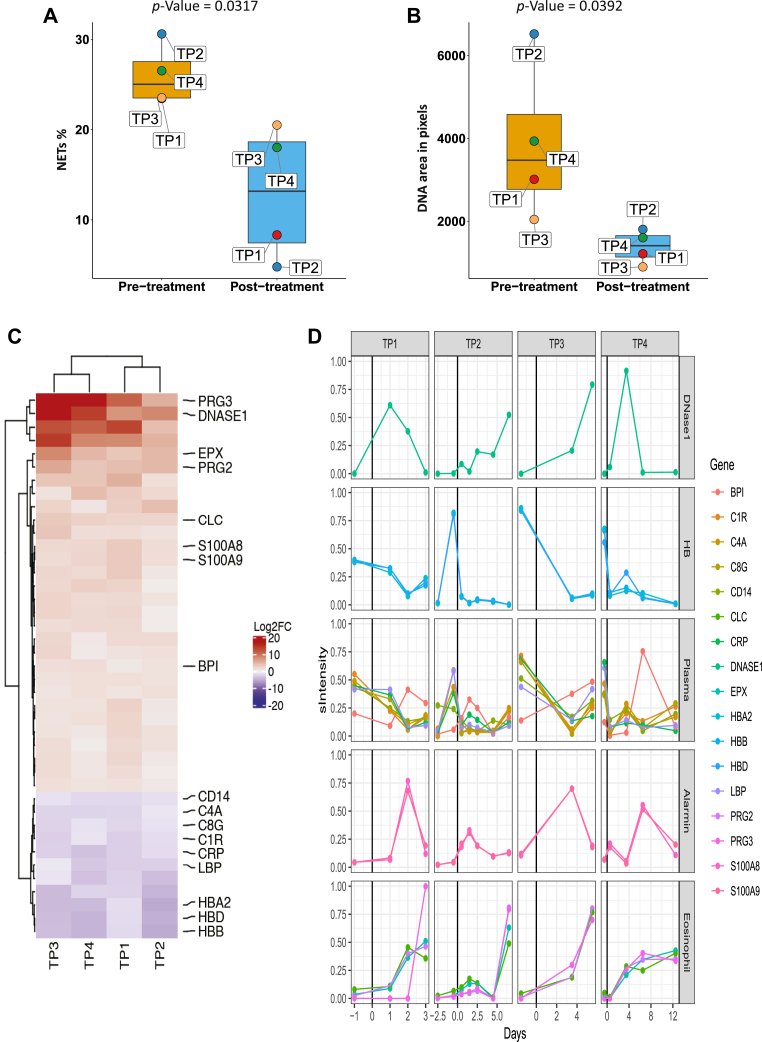
**Differential analysis of sputum protein abundance levels between COVID-19 patients before and after DNAse1 treatment.***A*, heat map of 45 proteins (*rows*) identified to change with rhDNase treatment by using *t* statistics (supplemental Fig. S5) in five patients (TP1–TP5). The mapped gene name for each protein is indicated to the *right*. The columns for each patient are the days relative to rhDNase treatment start (day 0) and are plotted as bar plots above the heat map. Colors in the heat map represent the scaled intensity per protein and patient (where maximum intensity is set to 1). *B*, individual plots of ten selected proteins from *A* (the scaled intensity is the same as in *A*). The *vertical black lines* in plots indicate day 0 (rhDNase treatment start). COVID-19, coronavirus disease 2019; rhDNase, recombinant human DNase I.

To study the effect of rhDNase on sputum NETs, we analyzed sputum NETs over time in four patients who produced sputum both before and after treatment using immunofluorescence and DIA-MS analysis. Representative images of sputum NETs before and after rhDNase treatment in each patient receiving HFNO therapy at treatment start are shown in Figure 4. Quantification of immunofluorescence against NE and DNA revealed that prior to rhDNase treatment (0.5–1 day), the patients had 23 to 31% NET-forming cells (Fig. 5*A*) and with DNA size of 2000 to 6500 px (Fig. 5*B*). At 2.5 to 5 days after initiation of rhDNase treatment, the proportion of NET forming cells (*p* = 0.0317) and NET DNA size (*p* = 0.0392) was significantly reduced in all patients. To assess sputum proteome composition, sputum samples prior to rhDNase treatment were compared with samples from after treatment, and 39 proteins were consistently found to be differentially abundant as judged by fold-change ratios and coefficient of variation (Fig. 5*C* and supplemental Table 2). As expected rhDNase (DNase I) was drastically elevated because of treatment. The levels of DNase I was however relatively variable between patients over time, which may reflect the variation in mucus turnover within individual patients. Some of the representative proteins that were more abundant after rhDNase treatment belong to eosinophils, basophils, and neutrophils. This indicates that the presence of the drug, and the ongoing cellular influx, coincides with improved respiratory function. A reduction in complement proteins, hemoglobin, lipopolysaccharide protein, and C-reactive protein was also associated with recovery. The variation in abundance over time of representative proteins in individual patients is presented in Figure 5*D*. All proteins are presented in a heat map, and their levels over time are listed in supplemental Figures S3 and S4.

### Proteomic Characterization of Blood Plasma in rhDNase-Treated Patients Reveals Reversal of Systemic Inflammation

**Fig. 6:**
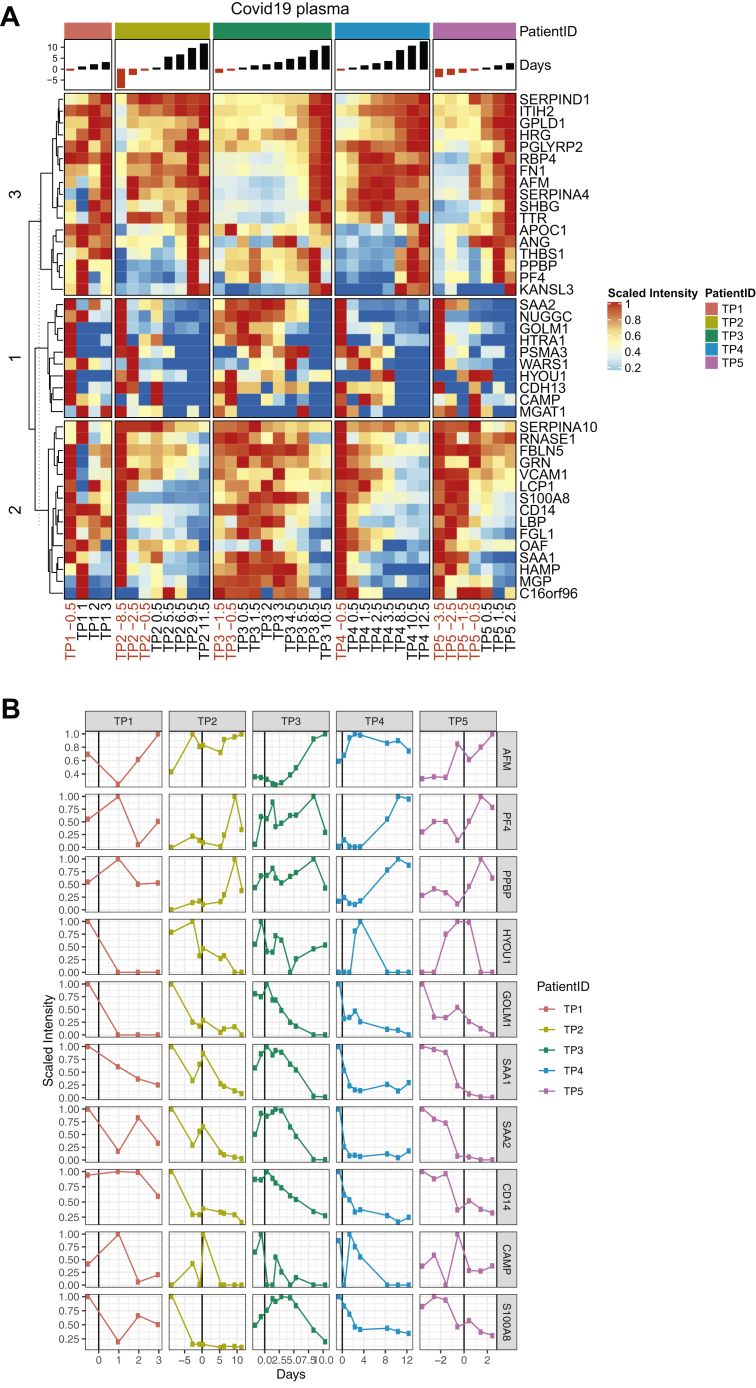
**Differential analysis of plasma protein abundance levels between COVID-19 patients before and after DNAse1 treatment.***A*, heat map of 45 proteins (*rows*) identified to change after DNAse1 treatment by using *t* test statistics (supplemental Fig. S5 in five patients [TP1–TP5]). The mapped gene name for each protein is indicated to the *right*. The columns for each patient are the days relative to DNAse1 treatment start (day 0) and are plotted as bar plots above the heat map. Colors in the heat map represent the scaled intensity per protein and patient (where maximum intensity is set to 1). *B*, individual plots of ten selected proteins from *A*) (the scaled intensity is the same as in *A*). The *vertical black lines* in plots indicate day 0 (DNAse1 treatment start). COVID-19, coronavirus disease 2019.

To evaluate systemic changes accompanying rhDNase treatment, blood plasma collected from all five patients was analyzed using DIA-MS. All blood plasma samples prior to DNase treatment were compared with post-treatment samples and are presented in a volcano plot revealing 45 statistically significant and differentially abundant proteins after correction for multiple hypothesis testing (supplemental Fig. S5 and Fig. 6*A*). rhDNase treatment and subsequent alleviation of ARDS increased the levels of 17 proteins, which included plasma proteins such as albumin and serine protease inhibitors, and platelet proteins (Fig. 6). A concomitant reduction in the amount of hypoxia upregulated protein 1, antiviral Golgi membrane protein 1, acute phase response proteins like serum amyloid proteins, and neutrophil-specific proteins like cathelicidin is also observed following treatment. Blood plasma levels of monocyte-associated protein CD14, which is involved in activating proinflammatory interleukin-6 signaling (49) and is known to be upregulated in severe COVID-19 (50), is also lowered after treatment. The variation in the levels of a few representative proteins in blood plasma over time are visualized in Figure 6*B*. These results also indicate that blood plasma following rhDNase treatment resembled healthy plasma as shown in supplemental Figures S6 and S7. In summary, sputum characterization revealed that recovery associated with rhDNase treatment was followed by reduced NETs, plasma proteins, and inflammatory proteins locally at the primary site of treatment. While systemically, blood plasma after treatment resembled healthy plasma proteome in terms of restored albumin, serine protease inhibitors and platelet proteins, and reduced acute phase proteins like serum amyloids, CD14, and lipopolysaccharide-binding protein.

## Discussion

In the work presented here, we show that the highly complex sputum proteome contains a high degree of NETs in various stages. Treatment of COVID-19 patients with rhDNase reduces the number of NETs and the inflammatory state in the sputum as well as alleviating the need for the administration of oxygen therapy. We found that the timing of sputum collection is variable because it depends on the patient's ability to expectorate it, and sputum composition can be highly variable on a daily basis in otherwise clinically stable patients because of fluctuating levels of inflammatory mediators (51). Excessive salivary contamination (52) could be another factor that adds to compositional variability of sputum. This is also true for the patients with COVID-19 included in our study and reflected in the MS analysis. None of the patients had bacterial superinfections, yet high numbers of neutrophils and NETs were present in the sputum of all patients, indicating that SARS-CoV2 itself can induce exorbitant neutrophil responses. Aging and coronaviruses can also reduce mucociliary clearance by altering cilia beating that may lead reduced clearance of mucus hypersecretions and cellular infiltrates (53, 54). Our data and previous reports (14, 55) suggest that cell-free DNA from NETs (and plausibly from apoptotic and necrotic cells) contributes to COVID-19 symptoms by thickening of mucus. Combined with the inability to clear mucus from the airways because of altered ciliary function, sputum production (56) is a major risk factor for the development of severe/critical COVID-19. The failure to clear thick sputum can obstruct airflow and impair gas exchange (33), eventually resulting in hypoxia-induced systemic inflammation (57). NETs alone also induce inflammation (58), and therefore, NETs could contribute to the cytokine storm found in severe COVID-19 (1). The procoagulant effect of NETs (26, 59) could contribute to the high risk of pulmonary thromboembolism in COVID-19 (60).

NETs have been described to be formed in response to SARS-CoV-2 (61); however, underlying host molecular mechanisms that govern NET formation in COVID-19 have not been described in detail. Increases in NET formation have been linked to severity and mortality in COVID-19 (29, 62). In line with previous findings, we also found increased NET formation prior to rhDNase therapy. This was gradually reversed following therapy, and recovery in all patients was associated with reduced NETs in the sputum. Further studies are needed to evaluate if lowered NETs in sputum are a marker of recovery in COVID-19.

rhDNase treatment reduced sputum NETs within 3 days; this indicates that rhDNase can penetrate sputum at clinically relevant doses and therefore could be a viable therapeutic to target NETs. DNase levels detected in the sputum during treatment varied over time and between patients, as observed in previous pharmacokinetic studies (63). Plasma levels of DNase were undetectable by MS before and after treatment start, likely because of absent or low concentrations of DNase in the blood. Previous studies have also found that aerosolized rhDNase administration did not significantly increase plasma DNase levels (64, 65). The patients were treated with Pulmozyme every 12 h over several days without any observable adverse effects, demonstrating that the drug was well tolerated. Overall, our data indicate that rhDNase pharmacokinetics and mode of action in COVID-19 sputum are likely similar to healthy and CF sputum, suggesting that doses and administration frequencies currently recommended for CF can be used in future studies of rhDNase in COVID-19. At the same time, there are still remnants of neutrophil proteins and NETs in the sputum after treatment, which indicates that higher doses of rhDNase potentially could more efficiently remove all DNA. Following rhDNase treatment, all five patients receiving COT or HFNO therapy did not deteriorate further and did not require intensive care unit care or a ventilator at any time. These patients were weaned from high-flow oxygen within 4 days of treatment start. Our results suggest that future clinical trials of rhDNase treatment should consider also including patients who are receiving oxygen therapy but do not yet require mechanical ventilation.

Although, in rhDNase-degraded NETs, we observed an increase in S100 A8 and 9 that could originate from neutrophils. This implies that neutrophil influx occurs during the process of recovery. Further studies are needed to examine the role of neutrophils after treatment. rhDNase treatment resulted in an increase in eosinophil proteins in sputum. Eosinopenia is known to be associated with severe COVID (66), and the reappearance of eosinophils could indicate recovery. We also observed lower amounts of complement proteins and hemoglobin with treatment. Together, these data indicate that rhDNase treatment caused a cellular reorganization and lowered the levels of plasma proteins in the airways.

Improved oxygenation after rhDNase treatment was also associated with the reorganization of the blood plasma proteome. Hypoalbuminemia and thrombocytopenia are associated with poor outcomes in COVID-19 (50, 67, 68). Following treatment, albumin and platelet proteins increased in concentrations indicating the reversal of acute conditions. Proteins that are protective against thrombosis like SERPIND1 or heparin cofactor II and inter-alfa-trypsin inhibitor heavy chain 2 were also upregulated. This may indicate a novel protective effect against coagulopathies commonly associated with COVID-19. Levels of C-reactive protein, a known marker of disease severity in COVID-19, were also reduced following rhDNase treatment (supplemental Fig. S6). Although this was not significant (*p* = 0.07), a clear trend toward reduction was observed. A similar pattern of reduction was observed for acute phase proteins, leukocyte proteins, and hypoxia-upregulated protein 1. Taken together, the differentially regulated protein patterns in blood plasma described in the article elucidate that improved oxygenation because of rhDNase therapy is associated with the lowering of hypoxemia, reduced inflammatory cells, and acute phase response proteins.

Limitations in study design include small sample size, the lack of randomization and controls, the lack of blinding at each analysis step, and the use of several concomitant medications such as chloroquine and low–molecular weight heparin. Apart from this, sampling sputum posed a significant challenge as it could not be performed regularly because of variation in sputum production by patients. In addition, samples for microscopy had to be processed immediately, as freezing could introduce artefacts. This made storage, processing, and performing batch analyses for microscopy very difficult. For these reasons, we could only examine sputum NETs in a small cohort of patients.

We believe that our study provides important information to complement and validate previous ELISA-based reports of NETs in COVID-19 (29). No evidence has so far been provided about the presence of NETs in the lungs and their involvement in causing ARDS in COVID-19. Two recent reports also found DNase to be beneficial in COVID-19 (69, 70). Weber *et al*. (69) described a combinatorial treatment with albuterol to be beneficial in severely ill COVID-19 that received mechanical ventilation and ECMO. Compared with our cohort, these patients were much sicker and three of five patients were weaned off ECMO within 8 days. Because of higher disease severity, patients continued to receive intensive care but showed steady signs of recovery. In contrast, patients in the cohort described by Okur *et al*. (70) are less sick, and DNase therapy is followed by improved oxygenation and also lowered GGO in lungs. The rhDNase treatment regimen of 2.5 mg rhDNase twice daily followed in both articles was similar to ours and without any treatment-associated toxicities. However, both reports did not address the presence of NETs through microscopy or ELISA-based methods in blood plasma, sputum, or in the airways of the patients. One of the strengths of this article is that we provide direct evidence showing the presence of intact NETs and NET-related proteins such as azurophilic granule–derived and citrullinated proteins in lungs, the main target organ of COVID-19. Second, we identified NETs as a source of extracellular DNA, which enhances sputum viscosity in severely ill patients with COVID-19 and that rhDNase treatment reduced NETs in sputa of recovered patients. Furthermore, using MS, we were able to monitor the reorganization of sputum and blood plasma proteome suggesting that reduced inflammatory proteins were associated with recovery after rhDNase treatment.

We realize that the reduction of inflammatory proteins, improved oxygenation, and recovery presented here may also be independent of the rhDNase therapy. Hence, further robust clinical trials are required to assess dosage, duration of therapy, and which patient groups presenting either mild or severe symptoms may benefit the most from rhDNase. Based on the data presented here, we are currently carrying out a phase 2 single-blinded, placebo-controlled, and randomized clinical trial (number: 2020-001849-39 in the EudraCT registry, ClinicalTrials.gov identifier: NCT04541979) to determine whether rhDNase reduces oxygen requirements in severe COVID-19. Nine trials have been registered so far.

Despite limitations, our study offers several novel findings related to COVID pathophysiology, treatment, and recovery. We have for the first time characterized the sputum proteome in patients with COVID-19 and reported that NETs are present in sputum of patients with severe COVID-19. NETs can cause impairment of respiratory function, and rhDNase treatment can improve respiratory function by degrading sputum NETs. This is associated with the reduction of inflammatory markers and plasma proteins in sputum and reduced acute phase proteins in blood plasma. NETs in the airways may, therefore, be an important therapeutic target in severe COVID-19. We believe that our study provides important information to aid with future clinical trials of rhDNase in COVID-19.

## Data availability

Data included in this article are available from the authors upon reasonable request. The MS proteomics data have been deposited to the ProteomeXchange Consortium *via* the PRIDE (refer: https://doi.org/10.1093/nar/gky1106) partner repository with the dataset identifier PXD021197.

## Supplemental data

This article contains supplemental data.

## Conflict of interest

A. L. and J. M. have a patent “Treatment of subjects suffering from COVID-19” pending. All authors declare that they have no conflicts of interest with the contents of this article.

## Supplemental Data

Supplemental Table S3

covid.sky

covid.sky.view

covid.skyl

covi-assay.blib

Citrullinated-peptides_psm

covid-assay.blib

covid.skyd
