# Supplementary material for: Proteome Profiling of Recombinant DNase Therapy in Reducing NETs and Aiding Recovery in COVID-19 Patients
Source: Mol Cell Proteomics. 2021 Jun 15;20:100113. doi: 10.1016/j.mcpro.2021.100113 (PMC8205261; doi:10.1016/j.mcpro.2021.100113)
Supplement: Supplemental files pdf [file mmc12.pdf]

Supplementary materials

Fig. S1. Citrullinated histones and granule-proteins from neutrophils are present in COVID-19 sputum

Fig. S2. DNase 1 degrades NETs in COVID-19 sputum

Table S1. Patient characteristics treated with rhDNase

Fig. S3. Heat map of sputum proteome after treatment with rhDNase.

Fig.S4. Sputum proteins over time per patient

Fig. S5. Volcano plot depicting plasma proteins significantly regulated after rhDNase treatment.

Fig. S6. Plasma proteins intensity plots per patient over time.

Fig. S7. Comparison of blood plasma from untreated, rhDNase-treated, and healthy individuals.

Supplementary methods – Detailed methods section

Tables Attached -

Supplementary table -1. List of proteins found in COVID-19 sputum

Supplementary table-2. Fold changes of proteins found in COVID-19 sputum before and after rhDNase treatment

Supplementary table-3. List of proteins found in blood plasma of COVID-19 patients

Supplementary figures

Fig. S1. Citrullinated histones and granule-proteins from neutrophils are present in COVID-19 sputum

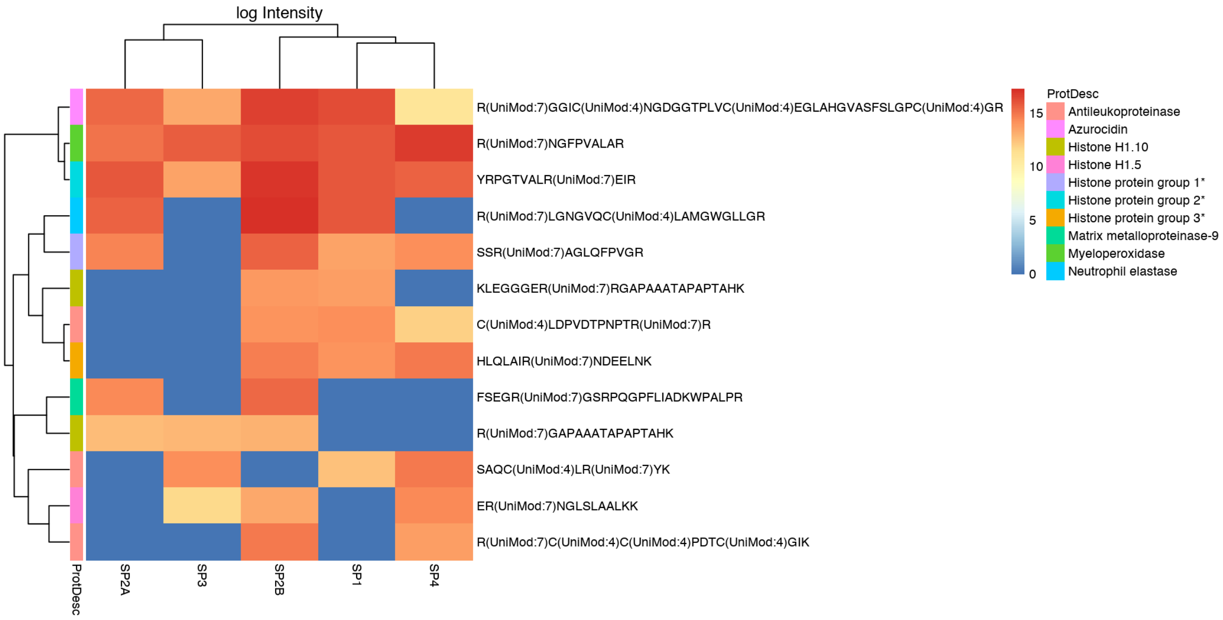

Identification of citrullinated peptides derived from Neutrophil NET proteins in Covid-19 lung sputum using DIA-MS.

A spectral library which included citrullinated peptides was built by searching pooled and fractionated Covid-19 lung sputum DDA runs with the variable modification +0.98 Da on arginine residues (UniMod:7) and up to three missed trypsin cleavages. This library was used for searching individual DIA-MS runs of lung sputum (n=5) from 4 Covid-19 patients. The peptide level results were filtered as follows: including NET proteins and including peptides with citrullinated modifications and finally excluding peptides with the citrullinated modification located in the C-terminal position of a peptide. The intensities of the filtered citrullinated peptides are shown in the heatmap. Each row represent a citrullinated peptide with modifications shown on the right side. The mapped proteins and genes are indicated with colours on the left side.

Fig. S2. DNase 1 degrades NETs in COVID-19 sputum

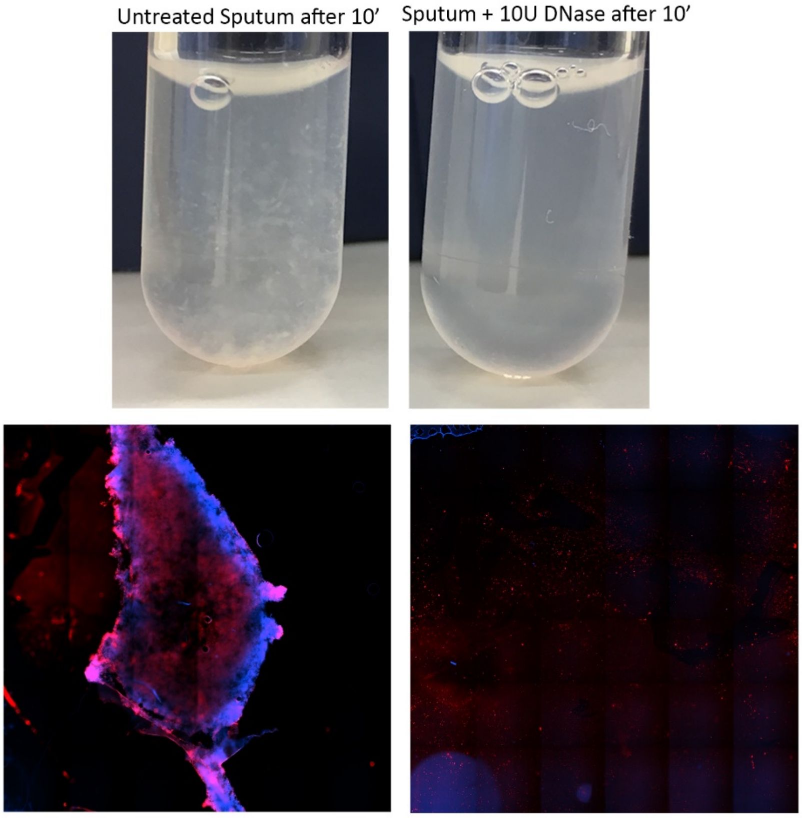

DNase degrades NETs in COVID-19 sputum and reduces viscosity. Sputum from a COVID-19 patient (n =1) was treated with +/- 10 units of DNase (Abcam) for 10 minutes at 37 degrees Celsius. This resulted in clarification of the sputum and reduced viscosity. Immunofluorescence against neutrophil elastase and DNA revealed that DNase treatment degraded NETs in COVID-19 sputum. Whereas large NETs persisted in untreated COVID-19 sputum.

Table S1. Baseline characteristics and clinical parameters of SARS-Cov2 positive patients prior to treatment with rhDNase

	TP1	TP2	TP3	TP4	TP5
**Baseline characteristics**					
Age (years)	50s	70s	60s	60s	60s
Gender	Male	Male	Male	Male	Female
Hospital stay (days)	7	26	16	19	8
**Concomitant medications**					
Antibiotics	Cefotaxime	Cefotaxime	Cefotaxime	Imipenem	Cefotaxime
Chloroquine phosphate(dose)	500mg BD	500mg BD	500mg OD	500mg BD	500mg OD
Anticoagulant		Enoxaparin	Enoxaparin		
Mucolytic		Acetylcysteine			
Anti-inflammatory		Betamethasone			
**Clinical parameters** (range before rhDNase treatment start)[1]					
Vital signs					
Temperature (°C)	35.8-36.9	36.2-38.1	37.9-40	36.8-40.3	35.1-37.7
Respiratory rate (breaths/min)	18-23	22-36	20-30	20-37	18-24
Pulse (beats/min)	82-98	67-100	70-110	85-120	75-97
Blood cell counts					
WBC ($x10^9$/L)	6.9-11.4	9.1-19.3	6.7-8.4	3.1-11.9	7-8.4
Neutrophils ($x10^9$/L)	6.3-11.1	8.4-17	5.3-7.1	2.7-2.8	5.4-7.2
Lymphocytes ($x10^9$/L)	0.3-0.4	0.2-1.2	0.5-0.9	0.3-0.3	0.7-1.1
Severity markers					
CRP (mg/L)	223-231	23-192	224-296	83-211	219-278
PCT (μg/L)	0.48	0.17-3.5	1.6		
LD (μkat/L)	7.3-7.3	6.6-13	4.2-5.4	1.2-9.7	
D-dimer (mg/L)	0.63	2.4-10	0.85-0.9	2-2	1.4-2.9
Ferritin (μg/L)	892-1042	649-3949	1535-1717	8748	1158-1342
Fibrinogen (g/L)	8.8-9	6.6-6.7	8.4		9-9
Triglycerides (mmol/L)	2.1-2.2	0.9-1.7	1.2.1.4	1.6	
NEWS	4-6	6-9	6-9	6-14	4-8
**Organ failures and biomarkers** (range before rhDNase treatment start)					
Total number of failing organs	1	3	2	2	2
Cognitive	no	no	no	no	no
Glasgow coma score	15-15	15-15	15-15	15-15	15-15
Liver	no	no	no	no	no

Creatinine (µmol/L)	67-82	138-318	129-155		62-150
Coagulopathy	no	no	no	no	no
INR	1.1	0.9-1	1	1.1	0.9-1.2
Platelets (x10^9/L)	260-314	283-650	214-234	134-162	208-293
Cardiovascular	no	**yes**	no	**yes**	no
Estimated MAP (mmHg)	82-102	68-90	73-90	63-93	78-98
Respiratory	**yes**	**yes**	**yes**	**yes**	**yes**
O_2 Saturation (%)	91-98	80-97	89-95	86-96	88-96
SpO_2/FiO_2	192-245	111-381	118-424	171-383	391-447
**rhDNase treatment**					
Treatment start (days from admission)	3.5	10.5	3.5	4.5	5
Treatment duration (days)	3	5	6	5	3

TP = treated patient; ACE2 = angiotensin converting enzyme-2; BD = bi-daily; OD = once daily; WBC = white blood cell count; CRP = C-reactive protein; PCT = procalcitonin; LD = lactate dehydrogenase; NEWS = national early warning score; ALT = alanine transaminase; AST = aspartate aminotransferase; INR = international normalized ratio; MAP = mean arterial pressure; SpO_2 = peripheral capillary oxygen saturation; = FiO_2 = fraction of inspired oxygen

[1]Where only one measurement was taken during this time, only one number is reported

Fig. S3. Heat map of sputum proteome after treatment with rhDNase.

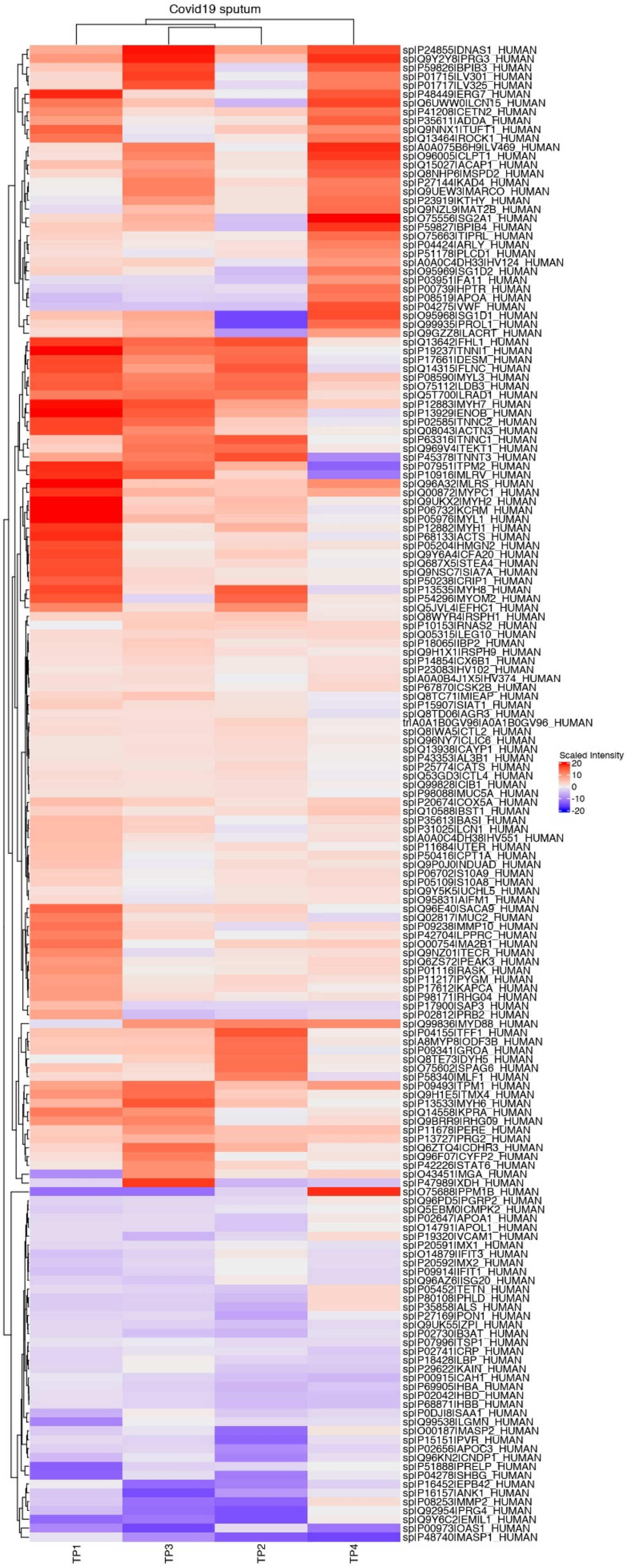

Covid19 sputum
sp|P24855|DNAS1_HUMAN
sp|Q9Y2Y8|PRG3_HUMAN
sp|P59826|BPIB3_HUMAN
sp|P01715|LV301_HUMAN
sp|P01717|LV325_HUMAN
sp|P48449|ERG7_HUMAN
sp|Q6UWW0|LCN15_HUMAN
sp|P41208|CETN2_HUMAN
sp|P35611|ADDA_HUMAN
sp|Q9NNX1|TUFT1_HUMAN
sp|Q13464|ROCK1_HUMAN
sp|A0A075B6H9|LV469_HUMAN
sp|O96005|CLPT1_HUMAN
sp|Q15027|ACAP1_HUMAN
sp|Q8NHP6|MSPD2_HUMAN
sp|P27144|KAD4_HUMAN
sp|Q9UEW3|MARCO_HUMAN
sp|P23919|KTHY_HUMAN
sp|Q9NZL9|MAT2B_HUMAN
sp|O75556|SG2A1_HUMAN
sp|P59827|BPIB4_HUMAN
sp|O75663|TIPRL_HUMAN
sp|P04424|ARLY_HUMAN
sp|P51178|PLCD1_HUMAN
sp|A0A0C4DH33|HV124_HUMAN
sp|O95969|SG1D2_HUMAN
sp|P03951|FA11_HUMAN
sp|P00739|HPTR_HUMAN
sp|P08519|APOA_HUMAN
sp|P04275|VWF_HUMAN
sp|O95968|SG1D1_HUMAN
sp|Q99935|PROL1_HUMAN
sp|Q9GZZ8|LACRT_HUMAN
sp|Q13642|FHL1_HUMAN
sp|P19237|TNNI1_HUMAN
sp|P17661|DESM_HUMAN
sp|Q14315|FLNC_HUMAN
sp|P08590|MYL3_HUMAN
sp|O75112|LDB3_HUMAN
sp|Q5T700|LRAD1_HUMAN
sp|P12883|MYH7_HUMAN
sp|P13929|ENOB_HUMAN
sp|P02585|TNNC2_HUMAN
sp|Q08043|ACTN3_HUMAN
sp|P63316|TNNC1_HUMAN
sp|Q969V4|TEKT1_HUMAN
sp|P45378|TNNT3_HUMAN
sp|P07951|TPM2_HUMAN
sp|P10916|MLRV_HUMAN
sp|Q96A32|MLRS_HUMAN
sp|Q00872|MYPC1_HUMAN
sp|Q9UKX2|MYH2_HUMAN
sp|P06732|KCRM_HUMAN
sp|P05976|MYL1_HUMAN
sp|P12882|MYH1_HUMAN
sp|P68133|ACTS_HUMAN
sp|P05204|HMGN2_HUMAN
sp|Q9Y6A4|CFA20_HUMAN
sp|Q687X5|STEA4_HUMAN
sp|Q9NSC7|SIA7A_HUMAN
sp|P50238|CRIP1_HUMAN
sp|P13535|MYH8_HUMAN
sp|P54296|MYOM2_HUMAN
sp|Q5JVL4|EFHC1_HUMAN
sp|Q8WYR4|RSPH1_HUMAN
sp|P10153|RNAS2_HUMAN
sp|Q05315|LEG10_HUMAN
sp|P18065|IBP2_HUMAN
sp|Q9H1X1|RSPH9_HUMAN
sp|P14854|CX6B1_HUMAN
sp|P23083|HV102_HUMAN
sp|A0A0B4J1X5|HV374_HUMAN
sp|P67870|CSK2B_HUMAN
sp|Q8TC71|MIEAP_HUMAN
sp|P15907|SIAT1_HUMAN
sp|Q8TD06|AGR3_HUMAN
tr|A0A1B0GV96|A0A1B0GV96_HUMAN
sp|Q8IWA5|CTL2_HUMAN
sp|Q96NY7|CLIC6_HUMAN
sp|Q13938|CAYP1_HUMAN
sp|P43353|AL3B1_HUMAN
sp|P25774|CATS_HUMAN
sp|Q53GD3|CTL4_HUMAN
sp|Q99828|CIB1_HUMAN
sp|P98088|MUC5A_HUMAN
sp|P20674|COX5A_HUMAN
sp|Q10588|BST1_HUMAN
sp|P35613|BASI_HUMAN
sp|P31025|LCN1_HUMAN
sp|A0A0C4DH38|HV551_HUMAN
sp|P11684|UTER_HUMAN
sp|P50416|CPT1A_HUMAN
sp|Q9P0J0|NDUAD_HUMAN
sp|P06702|S10A9_HUMAN
sp|P05109|S10A8_HUMAN
sp|Q9Y5K5|UCHL5_HUMAN
sp|O95831|AIFM1_HUMAN
sp|Q96E40|SACA9_HUMAN
sp|Q02817|MUC2_HUMAN
sp|P09238|MMP10_HUMAN
sp|P42704|LPPRC_HUMAN
sp|O00754|MA2B1_HUMAN
sp|Q9NZ01|TECR_HUMAN
sp|Q6ZS72|PEAK3_HUMAN
sp|P01116|RASK_HUMAN
sp|P11217|PYGM_HUMAN
sp|P17612|KAPCA_HUMAN
sp|P98171|RHG04_HUMAN
sp|P17900|SAP3_HUMAN
sp|P02812|PRB2_HUMAN
sp|Q99836|MYD88_HUMAN
sp|P04155|TFF1_HUMAN
sp|A8MYP8|ODF3B_HUMAN
sp|P09341|GROA_HUMAN
sp|Q8TE73|DYH5_HUMAN
sp|O75602|SPAG6_HUMAN
sp|P58340|MLF1_HUMAN
sp|P09493|TPM1_HUMAN
sp|Q9H1E5|TMX4_HUMAN
sp|P13533|MYH6_HUMAN
sp|Q14558|KPRA_HUMAN
sp|Q9BRR9|RHG09_HUMAN
sp|P11678|PERE_HUMAN
sp|P13727|PRG2_HUMAN
sp|Q6ZTQ4|CDHR3_HUMAN
sp|Q96F07|CYFP2_HUMAN
sp|P42226|STAT6_HUMAN
sp|O43451|MGA_HUMAN
sp|P47989|XDH_HUMAN
sp|O75688|PPM1B_HUMAN
sp|Q96PD5|PGRP2_HUMAN
sp|Q5EBM0|CMPK2_HUMAN
sp|P02647|APOA1_HUMAN
sp|O14791|APOL1_HUMAN
sp|P19320|VCAM1_HUMAN
sp|P20591|MX1_HUMAN
sp|O14879|IFIT3_HUMAN
sp|P20592|MX2_HUMAN
sp|P09914|IFIT1_HUMAN
sp|Q96AZ6|ISG20_HUMAN
sp|P05452|TETN_HUMAN
sp|P80108|PHLD_HUMAN
sp|P35858|ALS_HUMAN
sp|P27169|PON1_HUMAN
sp|Q9UK55|ZPI_HUMAN
sp|P02730|B3AT_HUMAN
sp|P07996|TSP1_HUMAN
sp|P02741|CRP_HUMAN
sp|P18428|LBP_HUMAN
sp|P29622|KAIN_HUMAN
sp|P00915|CAH1_HUMAN
sp|P69905|HBA_HUMAN
sp|P02042|HBD_HUMAN
sp|P68871|HBB_HUMAN
sp|P0DJI8|SAA1_HUMAN
sp|Q99538|LGMN_HUMAN
sp|O00187|MASP2_HUMAN
sp|P15151|PVR_HUMAN
sp|P02656|APOC3_HUMAN
sp|Q96KN2|CNDP1_HUMAN
sp|P51888|PRELP_HUMAN
sp|P04278|SHBG_HUMAN
sp|P16452|EPB42_HUMAN
sp|P16157|ANK1_HUMAN
sp|P08253|MMP2_HUMAN
sp|Q92954|PRG4_HUMAN
sp|Q9Y6C2|EMIL1_HUMAN
sp|P00973|OAS1_HUMAN
sp|P48740|MASP1_HUMAN
Scaled Intensity
20
10
0
-10
-20
TP1
TP3
TP2
TP4

Differential analysis of sputum protein abundance levels between covid-19 pateints pre- and post DNAse1 treatment. Heatmap of 170 proteins (rows) identified to change with DNAse1 treatment (TP1-TP4). The mapped gene name for each protein are indicated to the right. The columns for each patient are the days relative to DNAse1 treatment start (Day 0) and are plotted as barplots above the heatmap. Colours in the heatmap represent the the scaled intensity per protein and patient (where maximum intensity is set to 1).

Fig.S4. Sputum proteins over time per patient

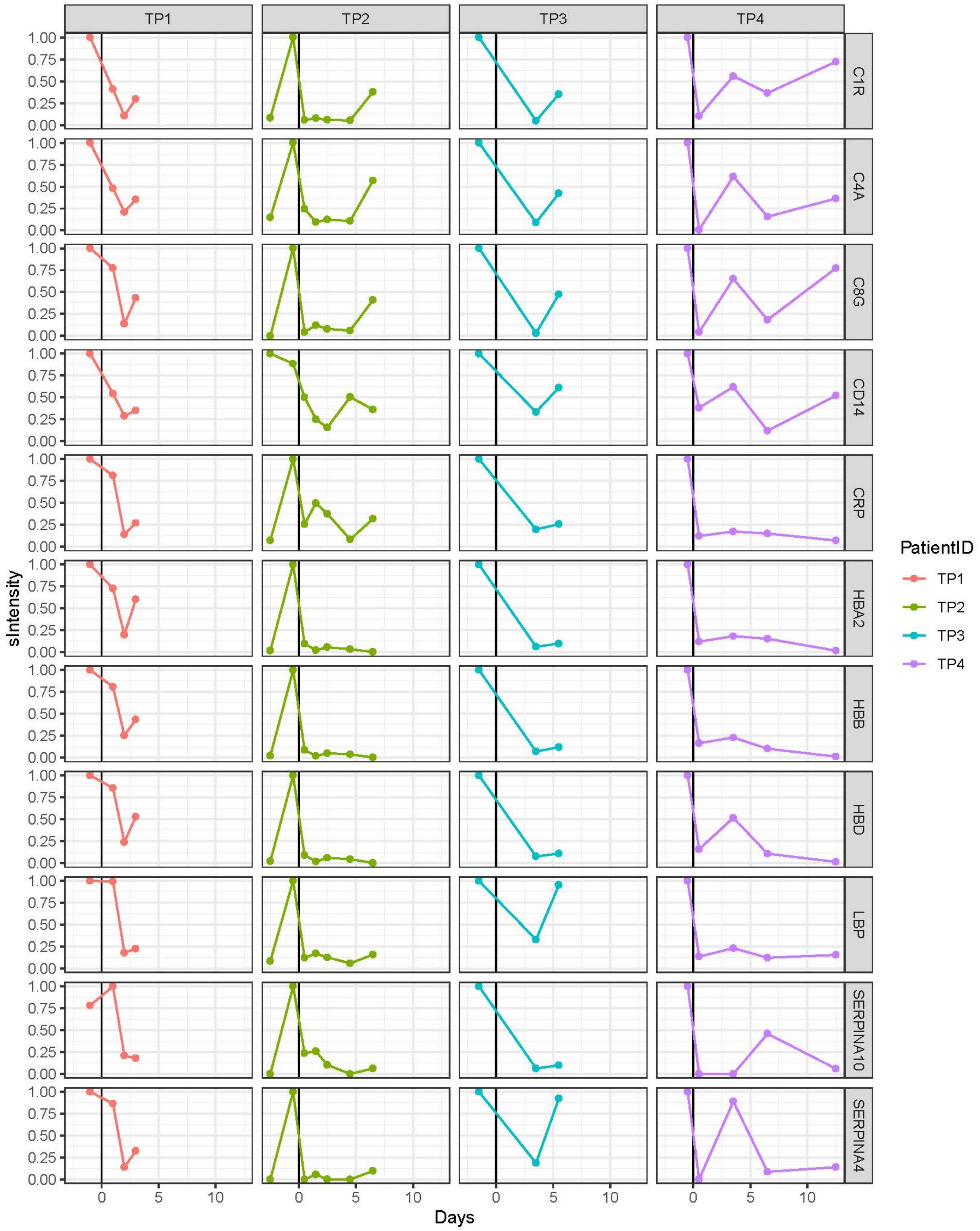

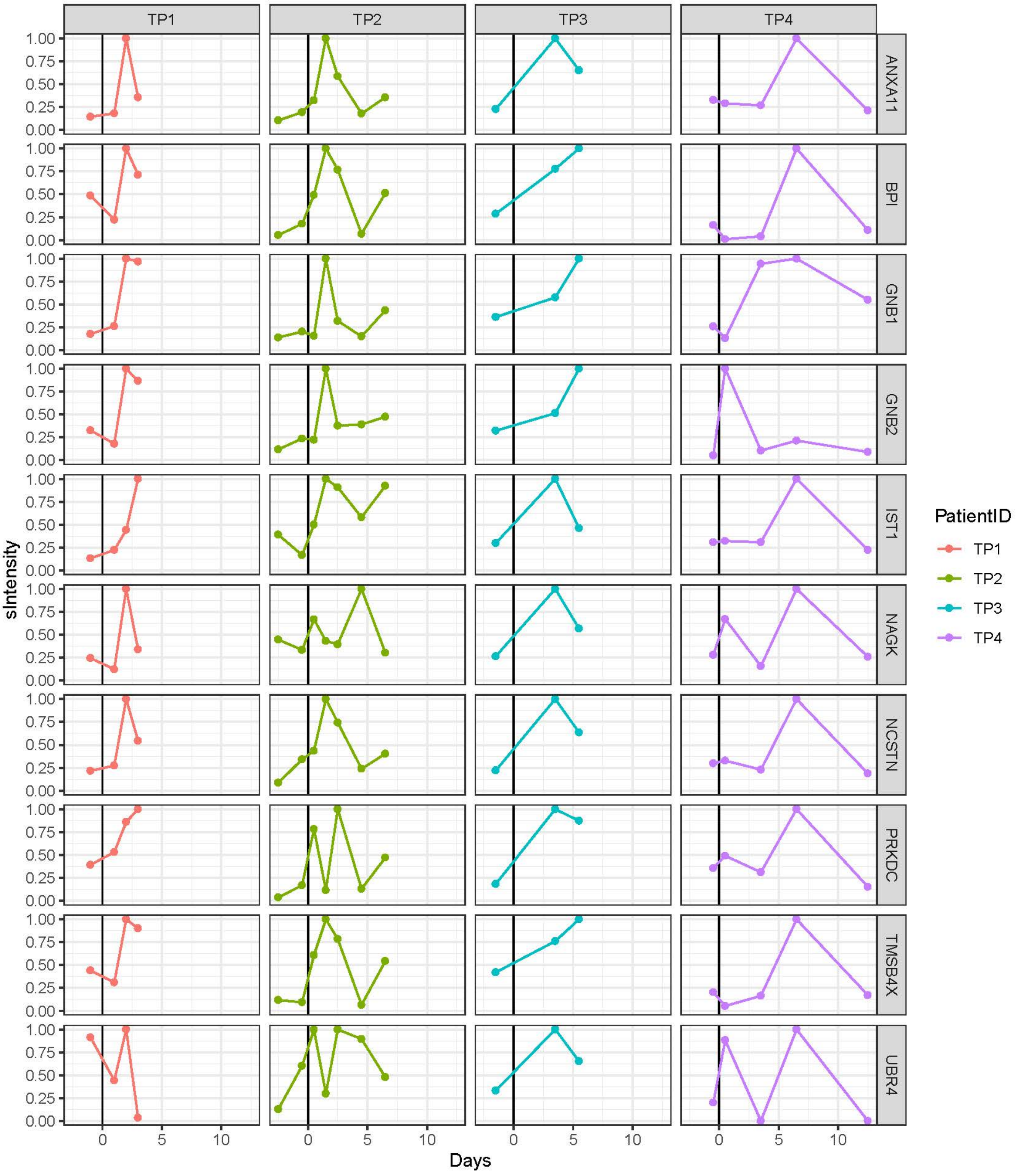

TP1
TP2
TP3
TP4
ANXA11
BPI
GNB1
GNB2
IST1
NAGK
NCSTN
PRKDC
TMSB4X
UBR4
sIntensity
Days
PatientID
TP1
TP2
TP3
TP4

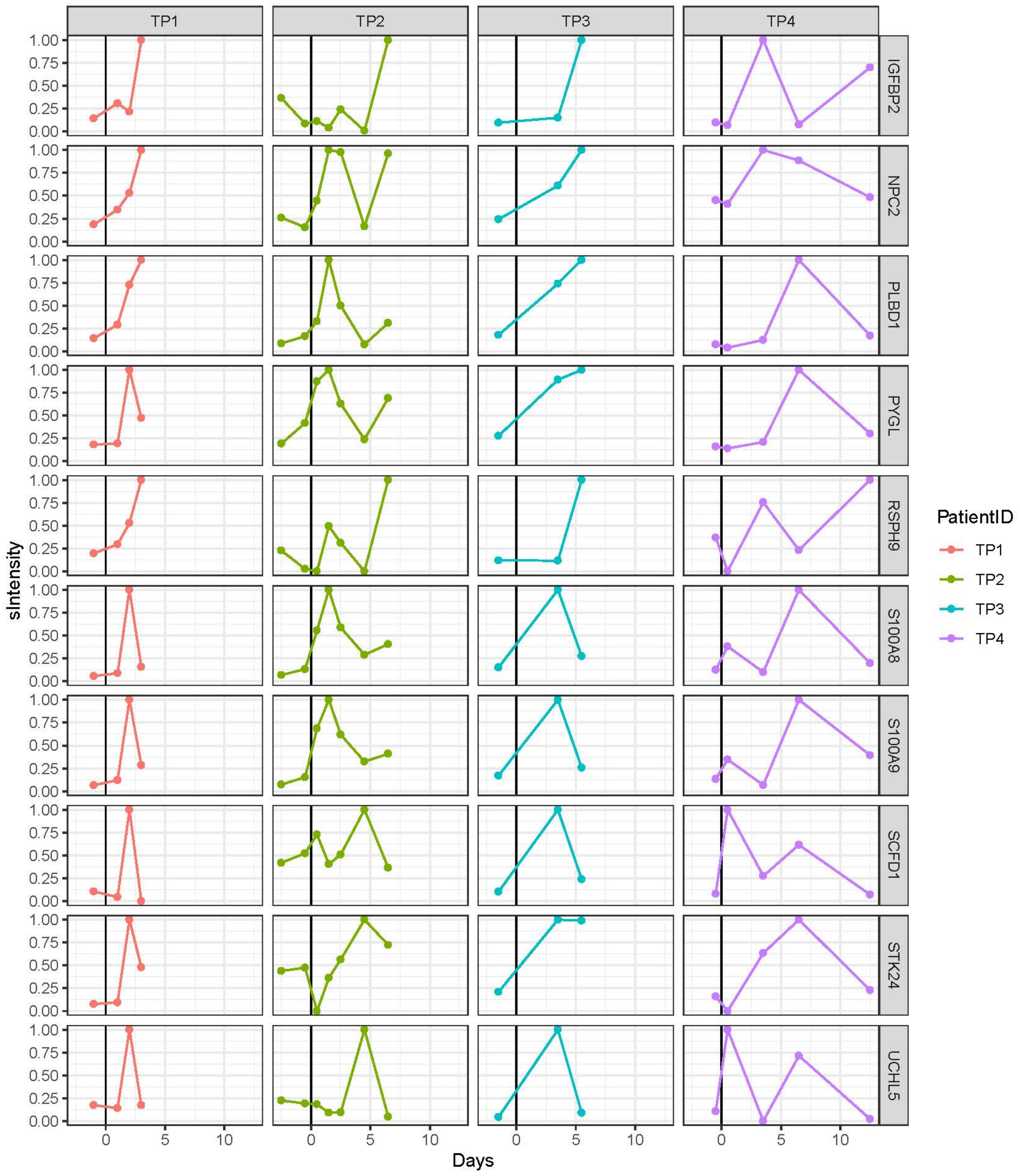

TP1
TP2
TP3
TP4
IGFBP2
NPC2
PLBD1
PYGL
RSPH9
S100A8
S100A9
SCFD1
STK24
UCHL5
sIntensity
Days
PatientID
TP1
TP2
TP3
TP4

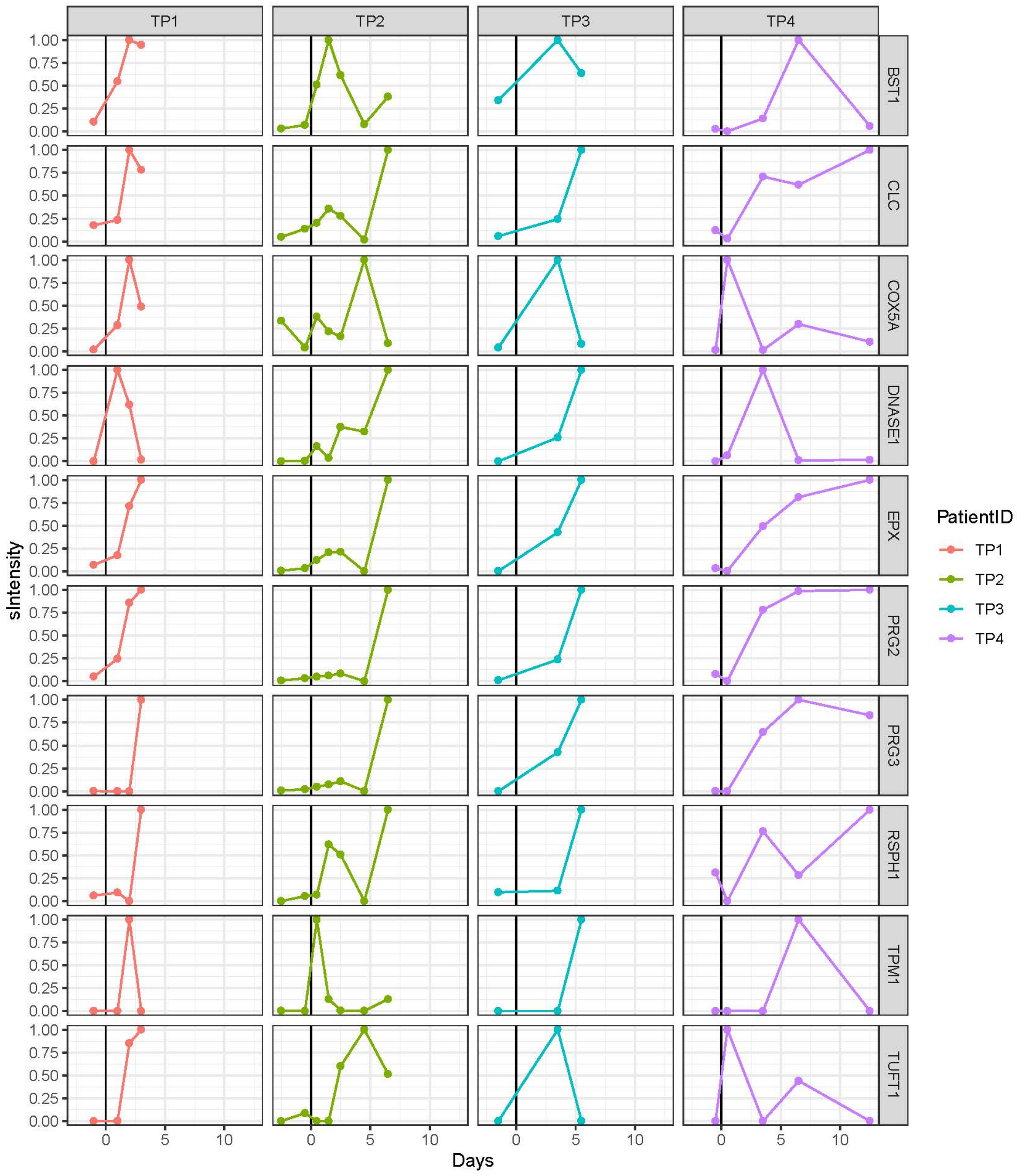

Individual plots of 10 selected proteins from a) (the scaled intensity is the same as in a). The vertical black lines in plots indicate Day 0 (DNAse1 treatment start).

Fig. S5. Volcano plot depicting plasma proteins significantly regulated after rhDNase treatment.

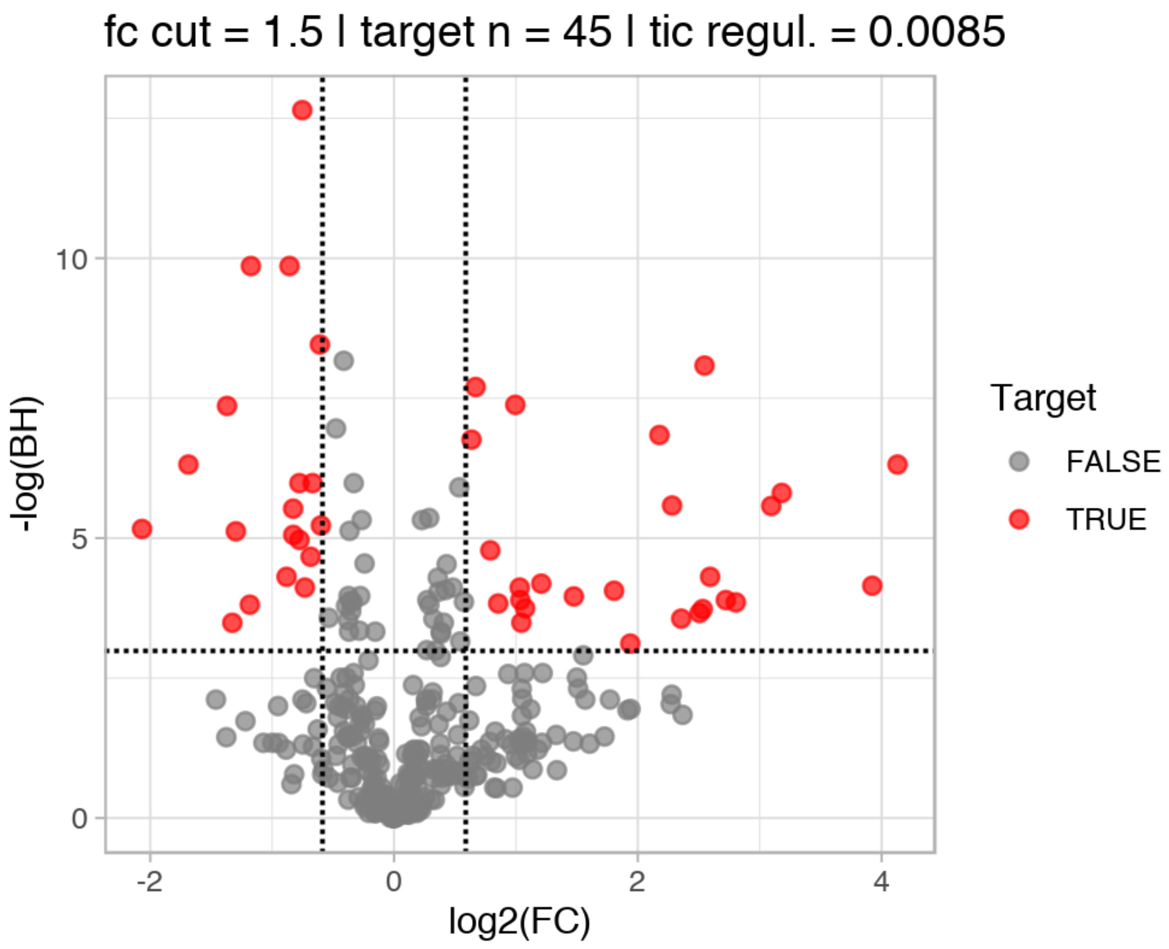

Volcano plot of plasma proteins of covid-19 pateints pre- versus post DNAse1 treatment. Vertical dotted lines are the foldchange cut offs (-1/1.5 and +1.5). Horizontal dotted line is the adjusted p-value cut off (0.05, Benjamini & Hochberg corrected).

Fig. S6. Plasma proteins intensity plots per patient over time.

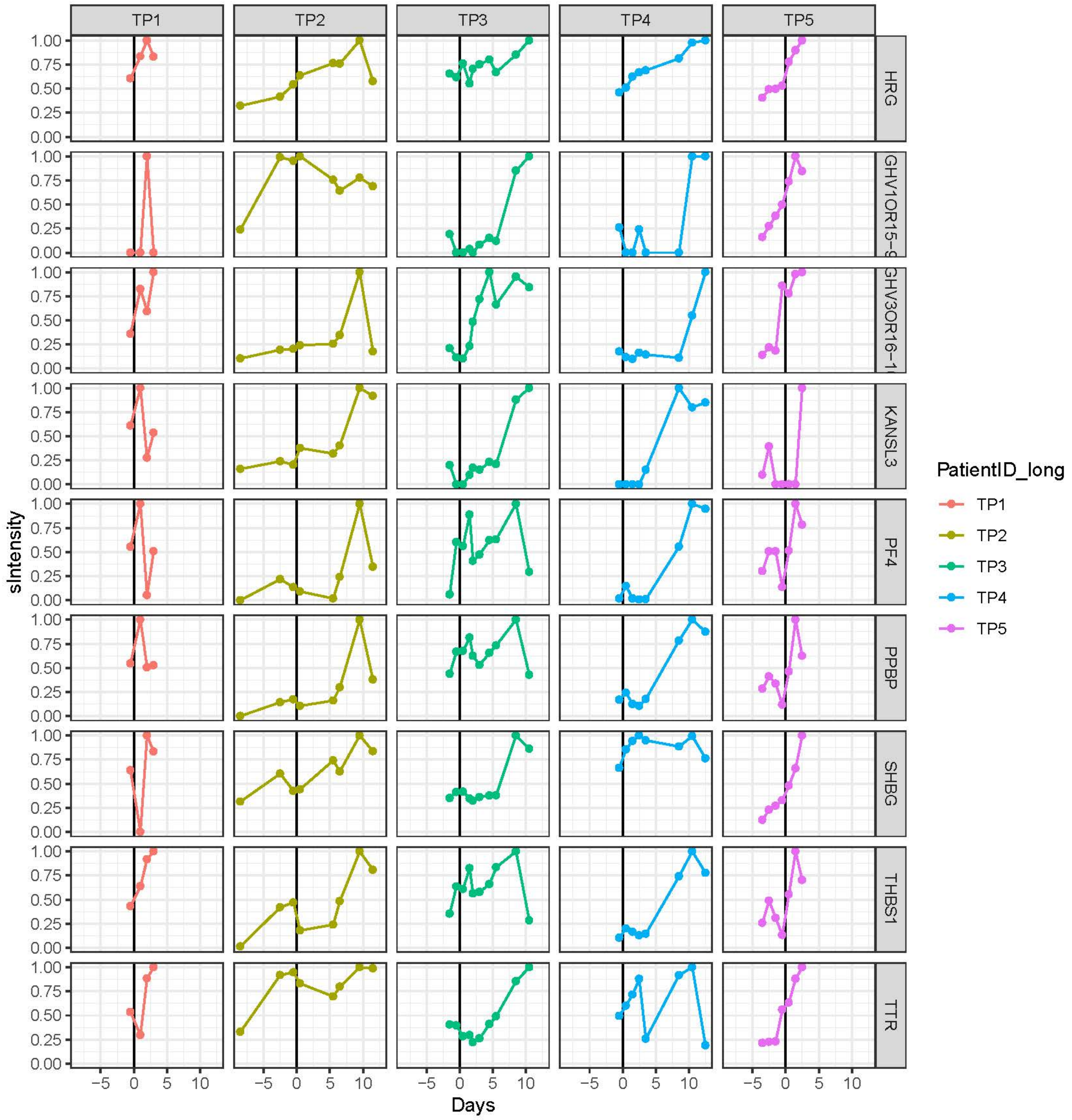

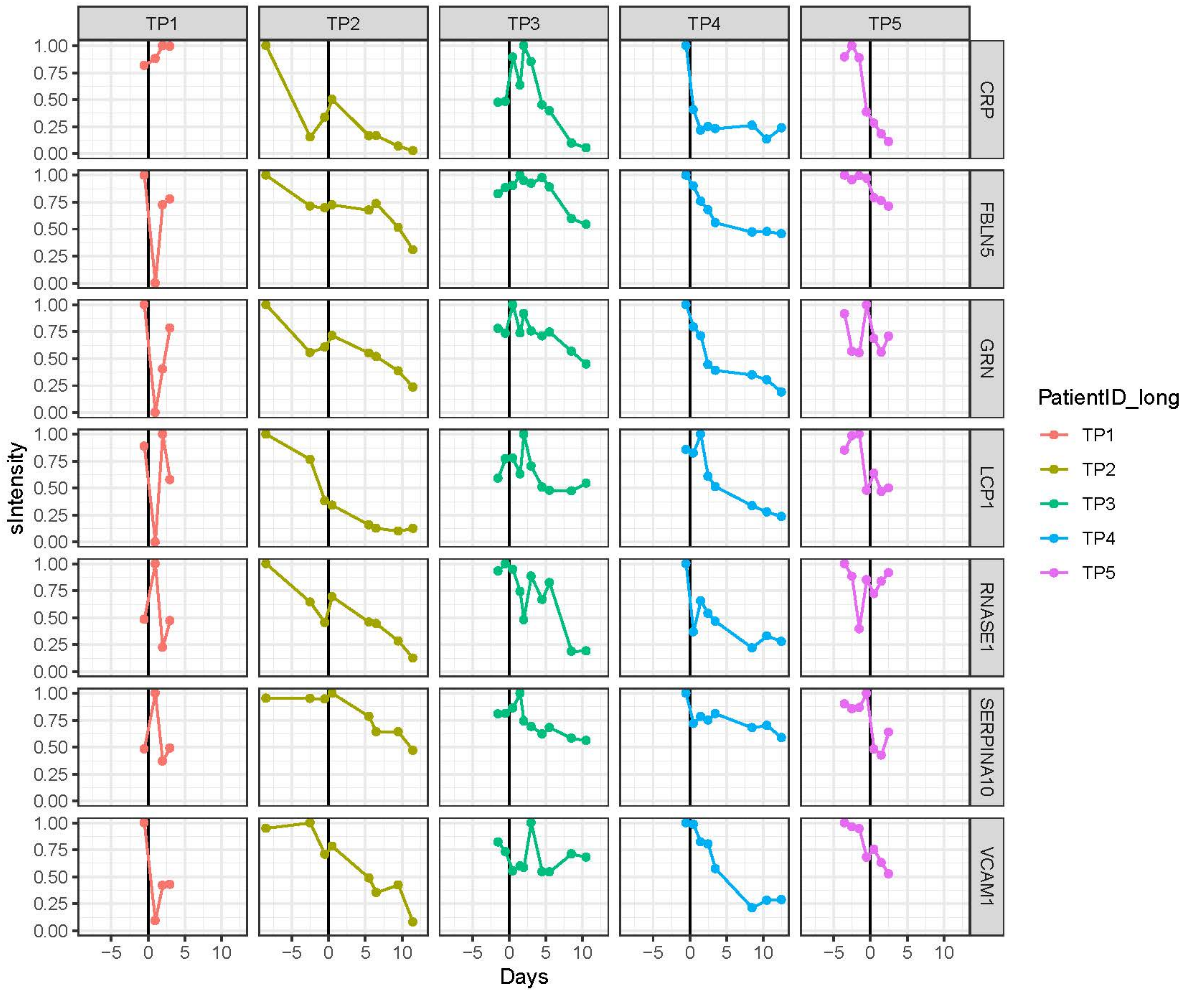
TP1
TP2
TP3
TP4
TP5
CRP
FBLN5
GRN
LCP1
RNASE1
SERPINA10
VCAM1
sIntensity
1.00
0.75
0.50
0.25
0.00
−5
0
5
10
Days
PatientID_long
TP1
TP2
TP3
TP4
TP5

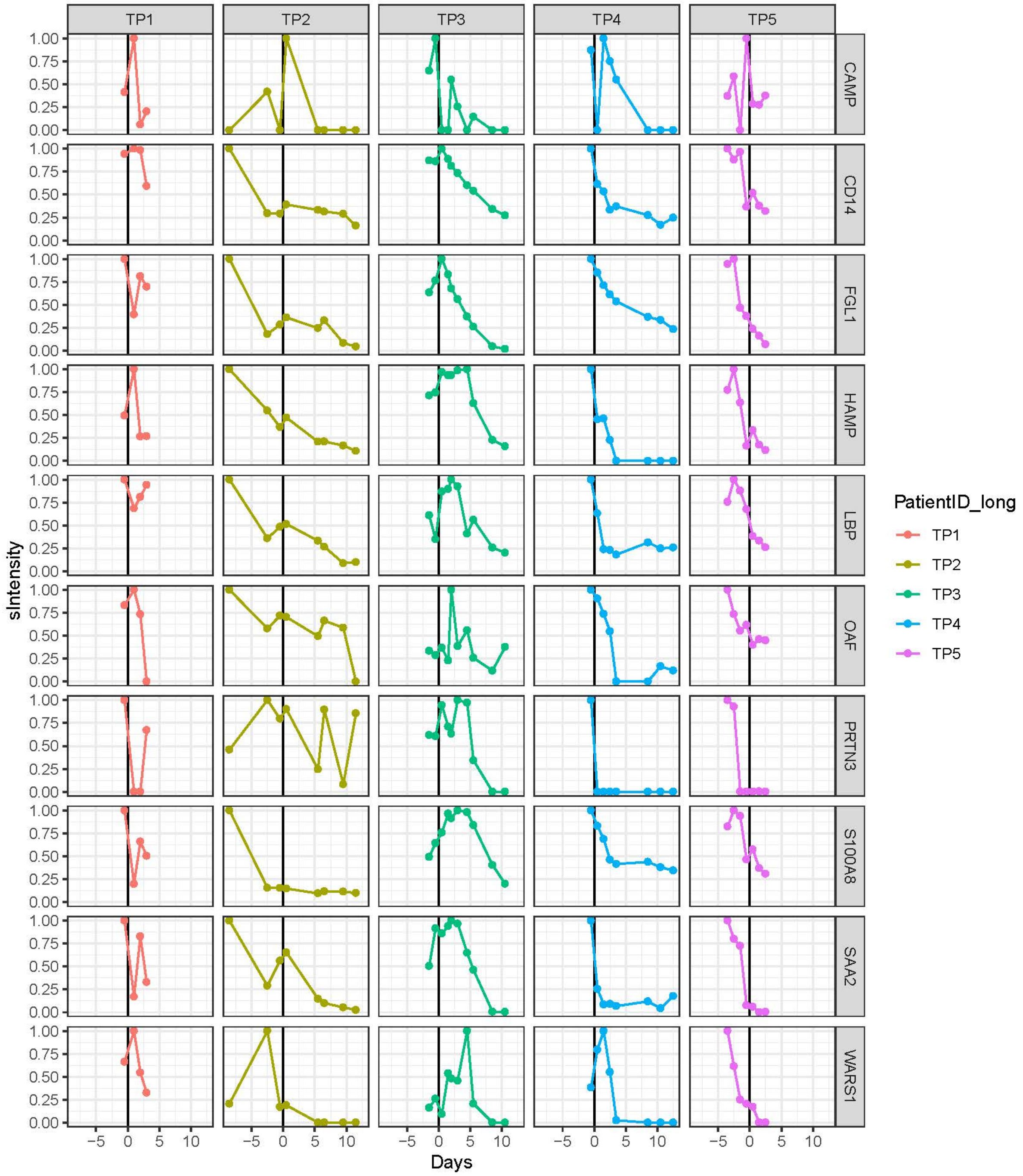

TP1
TP2
TP3
TP4
TP5
CAMP
CD14
FGL1
HAMP
LBP
OAF
PRTN3
S100A8
SAA2
WARS1
sIntensity
Days
PatientID_long

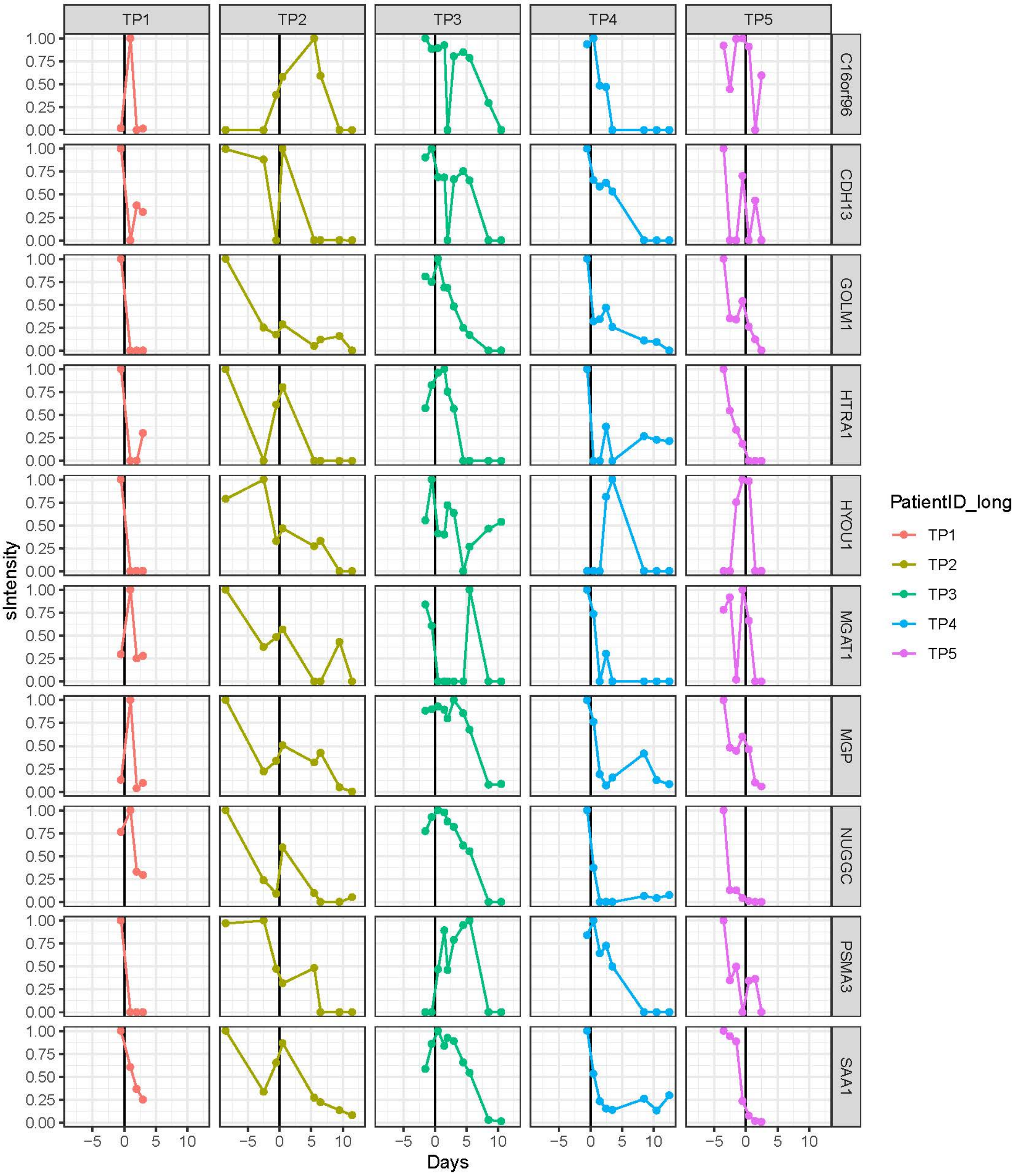
TP1
TP2
TP3
TP4
TP5
C16orf96
CDH13
GOLM1
HTRA1
HYOU1
MGAT1
MGP
NUGGC
PSMA3
SAA1
sIntensity
Days
PatientID_long

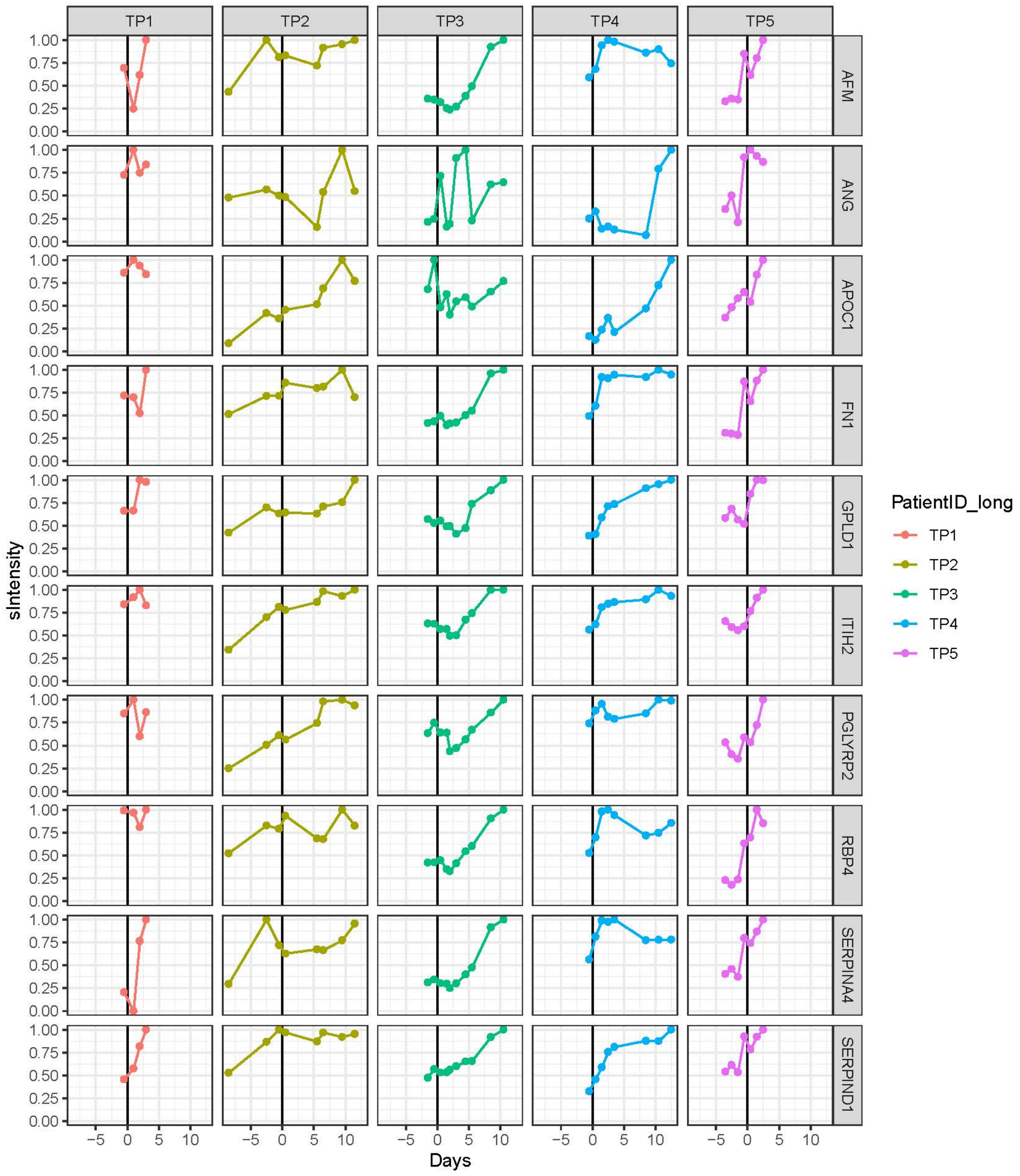

Individual plots of all proteins included in Figure 6. The vertical black lines in plots indicate Day 0 (DNAse1 treatment start).

Fig. S7. Comparison of blood plasma from untreated, rhDNase-treated, and healthy individuals.

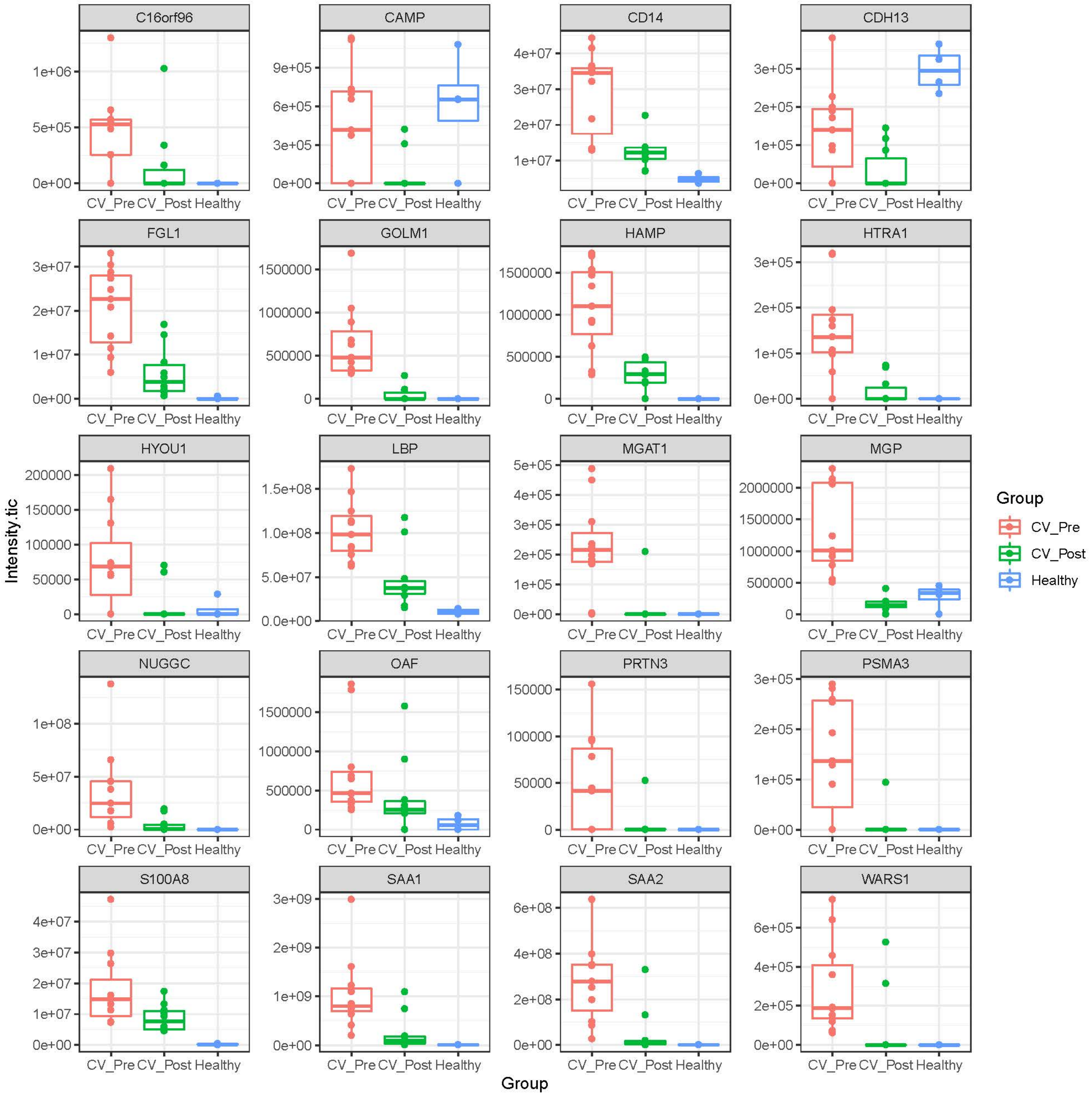

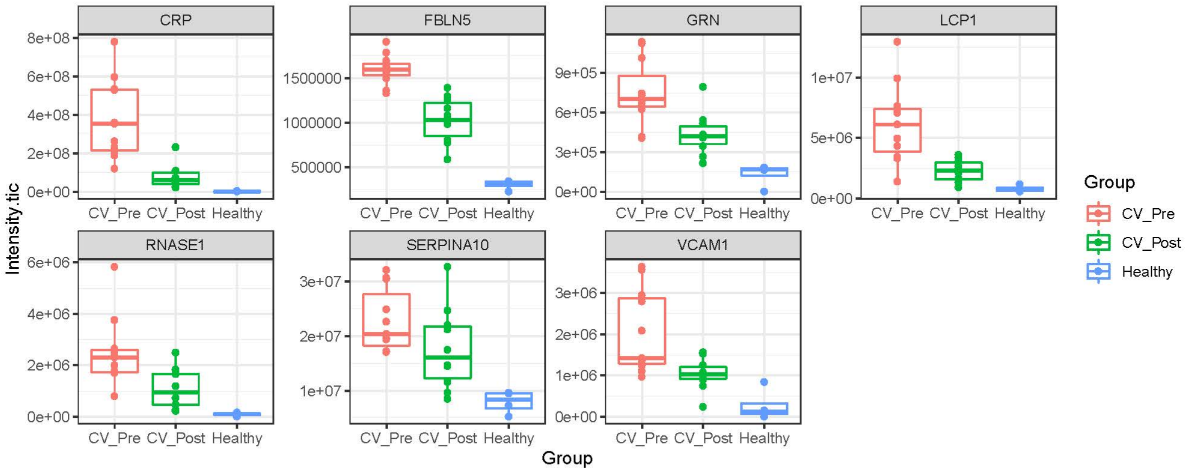
CRP
FBLN5
GRN
LCP1
RNASE1
SERPINA10
VCAM1
Intensity.tic
Group
CV_Pre CV_Post Healthy
Group
CV_Pre
CV_Post
Healthy

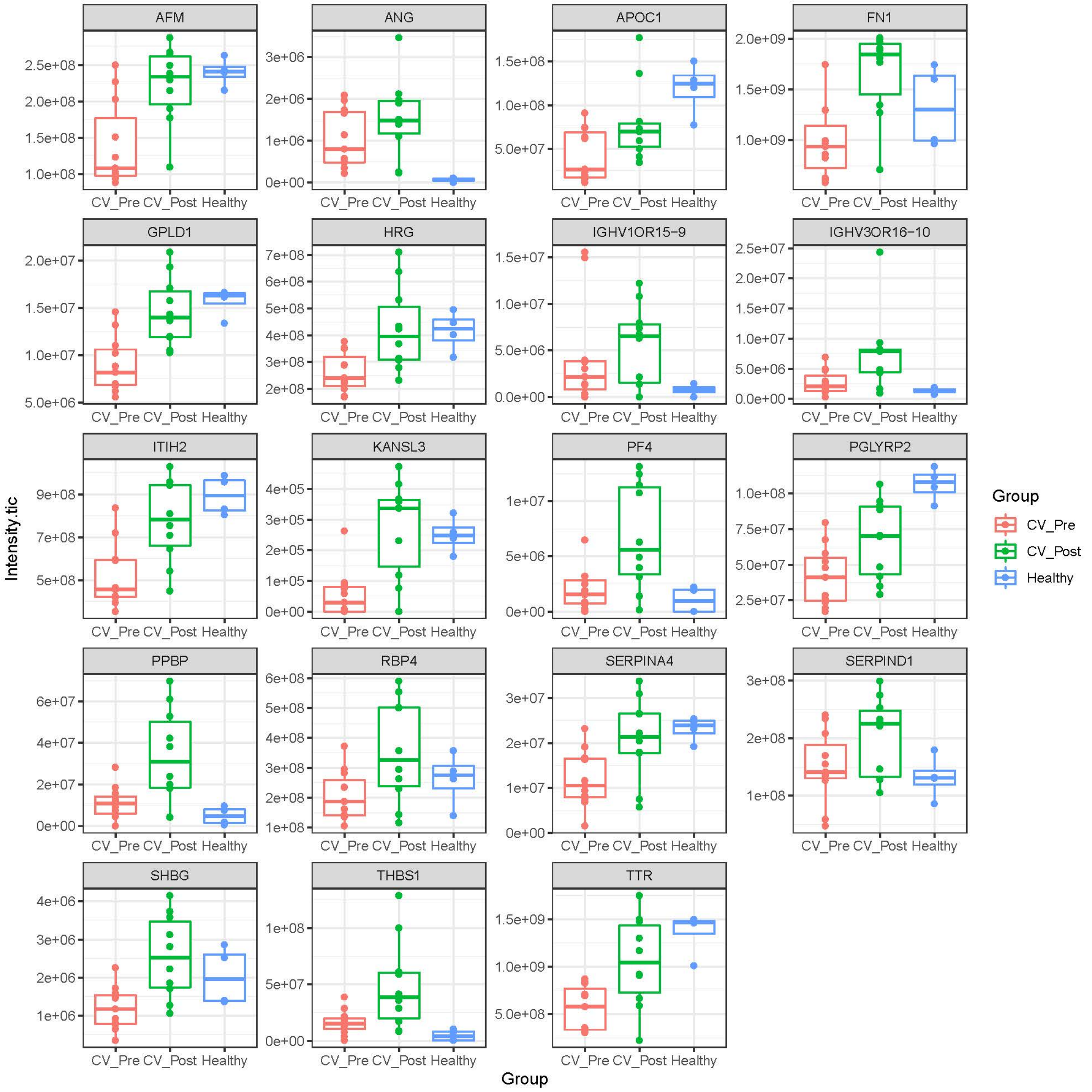
AFM
ANG
APOC1
FN1
GPLD1
HRG
IGHV1OR15−9
IGHV3OR16−10
ITIH2
KANSL3
PF4
PGLYRP2
PPBP
RBP4
SERPINA4
SERPIND1
SHBG
THBS1
TTR
Intensity.tic
Group
CV_Pre CV_Post Healthy
Group
CV_Pre
CV_Post
Healthy

Boxplots of all proteins included in the heat map presented in Figure 6 A compared with untreated (CV_Pre), rhDNase treated (CV_Post) and healthy blood plasma (Healthy).
